# Machine-learning assisted subclassification of glioblastoma by developing an endoplasmic reticulum stress-related methylation signature

**DOI:** 10.3389/fonc.2026.1750334

**Published:** 2026-04-22

**Authors:** Jinyi Zhao, Menglong Li, Xuemei Pu, Yanzhi Guo

**Affiliations:** College of Chemistry, Sichuan University, Chengdu, China

**Keywords:** DNA methylation, endoplasmic reticulum stress, glioblastoma, immune microenvironment, non-negative matrix factorization, random forest

## Abstract

**Introduction:**

Glioblastoma (GBM) is a highly aggressive brain tumor with significant heterogeneity, leading to poor prognosis and limited treatment options. Developing innovative molecular subtyping approaches is important for gaining deeper insights into disease pathogenesis and optimizing treatment strategies. DNA methylation has been implicated in the regulation of endoplasmic reticulum stress (ERS), which disrupts protein folding and activates the unfolded protein response (UPR), ultimately determining cellular survival or apoptotic outcomes.

**Methods:**

ERS-related DNA methylation profiles were integrated with non-negative matrix factorization (NMF) to establish a molecular classification framework for GBM. An ERS-based signature was further developed using recursive feature elimination with cross-validation (RFECV), and a random forest (RF) model was constructed for subtype prediction. The model was then applied to an external TCGA cohort for validation and downstream characterization.

**Results:**

The NMF-based framework stratified GBM patients into four distinct subtypes. The RF model achieved an accuracy of 92.4% in the independent test set. Application of the model to the TCGA cohort revealed distinct molecular and clinical characteristics across subtypes. In particular, Subtype 2 was associated with an immune-inflamed phenotype, lower tumor purity, and poorer prognosis. Connectivity Map (CMap) analysis further identified MEK inhibitors as preliminary candidate compounds for specific subtypes.

**Discussion:**

These findings support an association between ERS-related epigenetic modifications and GBM heterogeneity, and provide an epigenetic framework for refined molecular stratification and further exploration of subtype-related therapeutic strategies.

## 1 Introduction

As the most prevalent malignant primary brain tumor, glioblastoma (GBM) accounts for 47.6% of all intracranial and central nervous system (CNS) malignancies (1). Its highly aggressive behavior and pronounced heterogeneity contribute to poor survival, making GBM a major challenge in neuro-oncology research.

Molecular subtyping is pivotal for understanding the GBM’s complicated mechanism. The landmark transcriptomic classification by Verhaak et al. (2), including Proneural, Neural, Classical and Mesenchymal subtypes, has established a foundational framework for tumor stratification based on gene expression patterns. Similarly, Wang et al. (3) proposed a lineage-based classification in which Type I tumors are associated with EGFR amplification and neural stem cell features, whereas Type II tumors exhibit oligodendrocyte lineage characteristics and high ERBB3 expression. In parallel, DNA methylation-based classification has provided another important dimension for glioma stratification. Ceccarelli et al. (4) identified six DNA methylation subtypes across diffuse gliomas, including LGm4, LGm5, and LGm6, which are enriched in IDH-wild-type and GBM-related molecular states. More broadly, Capper et al. (5) developed a methylation-based classifier for CNS tumors that has shown strong diagnostic utility, further highlighting the robustness of methylation profiling for tumor classification. Together, these studies have greatly advanced the molecular classification of GBM and highlighted the value of integrating diverse molecular layers for tumor stratification. However, given the substantial biological heterogeneity of GBM, further subclassification from specific mechanistic perspectives may still provide additional insight for precision medicine.

Tumor heterogeneity arises from molecular, biological, and genetic alterations acquired during tumor evolution and is reflected in differences in genomic, transcriptomic, proteomic, and epigenetic features across patients and even across regions within the same tumor. Such heterogeneity influences the growth, invasion and drug sensitivity of tumor cells (6, 7). Among these different molecular layers, epigenetic alterations, especially DNA methylation, have been increasingly recognized as important regulators of cancer initiation and progression. DNA methylation involves the addition of methyl groups to specific DNA sites without altering the base sequence, and it regulates gene expression by affecting the binding of transcription factors, thereby influencing cellular functions such as DNA repair (8, 9). The cancer methylome reflects both the acquired epigenetic alterations and the cellular origins of tumor cells. Importantly, DNA methylation patterns are highly stable and reproducible, even in low-quality or small-quantity samples (5). Therefore, investigating DNA methylation patterns may help refine molecular subclassification of GBM by capturing a biologically meaningful layer of heterogeneity that complements existing classification frameworks.

The endoplasmic reticulum (ER) serves as the primary site for protein synthesis, post-translational modification and vesicular transport, with its homeostatic regulation being critical for maintaining cellular proteostasis and function (10). Accumulation of unfolded or misfolded proteins triggers endoplasmic reticulum stress (ERS), activating the unfolded protein response (UPR) through three major pathways, including IRE1, PERK and ATF6 (11, 12). The UPR aims to restore ER balance by enhancing degradation of misfolded proteins and reducing mRNA translation. When this protective response fails, the UPR activates apoptotic signaling pathways. In GBM, tumor cells proliferate in a hypoxic and immunosuppressive microenvironment with aberrant vascularization and a dysfunctional blood-tumor barrier, which would frequently induce persistent ER stress. Although ER stress responses also occur in normal cells, cancer cells can exploit sustained UPR activation to enhance protein quality control and support long-term survival. Therefore, this aberrant signaling is considered a key factor affecting tumor progression and treatment response. For example, Auf et al. have found that inhibiting IRE1α in a murine GBM model significantly suppressed tumor growth and reduced angiogenesis and tumor perfusion (13).

Growing evidence suggests that ERS is not only driven by environmental stress but also regulated by epigenetic mechanisms, including aberrant DNA methylation and histone modifications in the promoter regions of ERS-related genes (14). As an illustrative case, Le et al. (15) have reported that ZDHHC1, located on chromosome 16q22.1, functions as a tumor suppressor gene. Promoter methylation of this gene leads to its silencing in multiple tumor cells, and restoring its expression can promote cancer cell apoptosis by increasing ER stress. These findings suggest that complex regulatory interactions exist between ERS and DNA methylation and that such interactions may contribute to interpatient biological differences. Therefore, investigating ERS-related DNA methylation patterns may provide a rational strategy for identifying novel molecular subtypes of GBM.

**Figure 1:**
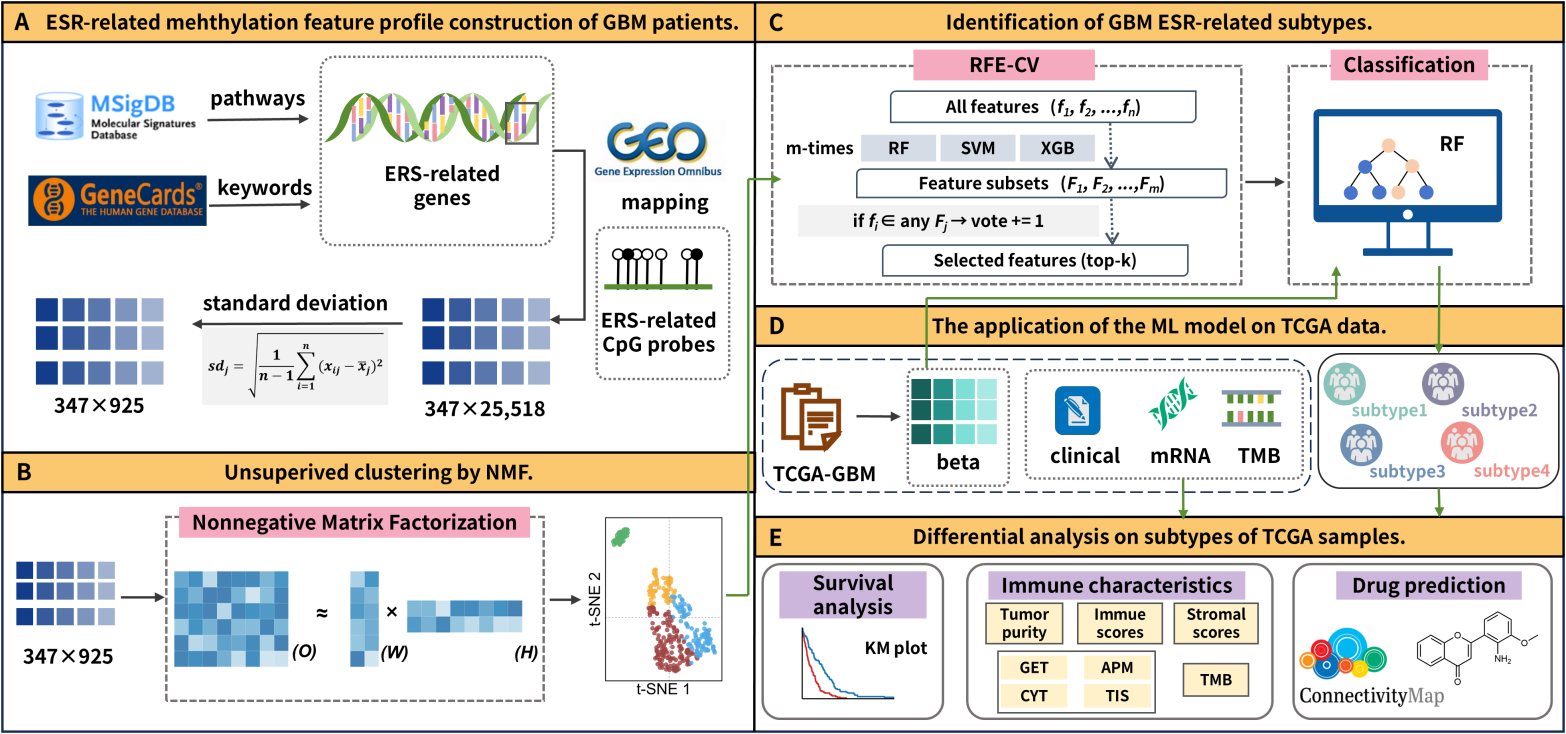
The flowchart of the research. **(A)** ESR-related methylation feature profile construction of GBM patients. **(B)** Unsupervised clustering by NMF. **(C)** Identification of GBM ESR-related subtypes. **(D)** The application of the ML model on TCGA data. **(E)** Differential analysis on subtypes of TCGA samples.

In this study, we developed a novel classification framework for GBM based on DNA methylation profiles of ERS-associated genes and further evaluated its biological significance through comprehensive analyses. **Figure 1** shows the flowchart of this research. Firstly, ERS-related methylation feature profiles were constructed for GBM patients (**Figure 1A**). Here ERS-related gene features were derived from MSigDB and GeneCards, with methylation and transcriptomic data obtained from GEO. Subsequently, we selected the most variable CpG probes and performed unsupervised clustering using non-negative matrix factorization (NMF) to identify four novel molecular subtypes (**Figure 1B**). On this basis, we employed recursive feature elimination with cross-validation (RFECV), combined with three distinct machine-learning (ML) approaches to construct a classification model that enables robust stratification of samples (**Figure 1C**). The established model was then applied to classify samples in the TCGA cohort into the four ERS-related subtypes (**Figure 1D**). To validate the proposed subtyping system, differential analyses were performed across the identified subtypes. Specifically, survival analysis, immune microenvironment characterization, and drug prediction were conducted to elucidate their biological significance and potential clinical utility (**Figure 1E**).

## 2 Material and methods

### 2.1 Selection of ERS-related genes

In our study, genes related to ER stress were selected from GeneCards (16) (http://www.genecards.org) and MSigDB (17) (https://www.gsea-msigdb.org/gsea/msigdb) database to construct an ERS-related gene set. In the GeneCards database, ERS-related genes were identified using the keyword “endoplasmic reticulum stress”, applying a relevance score threshold of ≥7 to ensure the selection of highly-correlated genes. Additionally, to ensure comprehensive coverage, ERS-related genes were extracted from MSigDB database.

As a result, a total of 1,319 ERS-related genes meeting the criteria were extracted from GeneCards, while 265 genes were obtained from MSigDB. After taking the union of the two gene sets, a final list of 1,380 ERS-related genes was generated (**Supplementary Table 1**).

### 2.2 GBM methylation data collection

A complete overview of data collection and preprocessing is provided in **Supplementary Figure 1**. This study used the DNA methylation dataset GSE90496 from GEO database as the discovery cohort for subtype identification and model construction. Raw IDAT files were imported into R using the minfi package, and Illumina-style preprocessing was performed with the custom function MNPpreprocessIllumina. Because the raw GSE90496 dataset contains multiple CNS tumor types, only samples annotated as GBM were retained for downstream analysis, resulting in a GEO discovery cohort of 347 cases. Probes were filtered by removing ambiguous probes, EPIC-specific probes, SNP-related probes, sex chromosome probes, and probes annotated as rs/ch. After log2 transformation, batch effects related to sample material type, including FFPE and frozen specimens, were corrected separately for methylated and unmethylated intensities using the removeBatchEffect function in the limma package. Adjusted β values were then recalculated from the batch-corrected methylated and unmethylated intensities. The final GEO discovery matrix comprised 347 GBM samples and 428,799 CpG probes. Genomic location and chromosome annotation information for these probes were obtained from the Illumina HumanMethylation450 BeadChip annotation platform.

Additionally, we employed the TCGA-GBM methylation dataset as an independent external validation set. The processed β-value matrix was downloaded from the UCSC Xena platform (https://xenabrowser.net). The dataset includes 153 GBM patients, covering 485,777 CpG sites. For quality control, only primary tumor samples with the sample-vial code 01A in the TCGA barcode were retained, yielding 126 cases as the external samples for the subtype identification. Among these, 123 cases with available overall survival (OS) information were included in the survival analysis. The available matched mRNA expression data of 48 cases were used for transcriptomic analyses. Tumor mutation burden (TMB) data were available for 118 cases that are for mutation-related analyses. Other relevant clinical information was also obtained from TCGA for molecular and clinical characterization of the identified subtypes.

### 2.3 NMF

The fundamental principle of NMF is to decompose a non-negative matrix into two non-negative matrices, thereby extracting the latent features embedded within the data. Given a non-negative matrix V of size m×n, NMF aims to identify two non-negative matrices, W (m×k) and H (k×n), such that V≈W*H. Here, k is smaller than both m and n and represents the number of latent features underlying the data. By appropriately selecting k, NMF can effectively capture the principal features of the data while simultaneously reducing its dimensionality (18). Owing to its ability to represent complex data as additive non-negative components, NMF has been widely employed in various bioinformatics applications, including gene expression analysis and single-cell sequencing (19).

In our study, NMF was applied to the methylation β-value matrix after preprocessing and probe filtering, using the subset of highly variable CpG probes selected for clustering analysis. The optimal number of clusters (k) was determined based on clustering quality evaluation metrics. Brunet et al. (20) suggested assessing clustering stability using the cophenetic correlation coefficient and recommended selecting the smallest k value at which the cophenetic coefficient begins to decline as the optimal number of clusters. NMF was implemented using the NMF package in R, with candidate ranks ranging from 2 to 10. For each rank, the algorithm was repeated 50 times with random initializations to improve clustering stability. A fixed random seed of 42 was set to ensure reproducibility. Clustering robustness was evaluated based on the consensus matrix and several stability metrics, including the cophenetic correlation coefficient, silhouette consensus score, dispersion, and explained variance. The final clustering solution was selected by jointly considering these stability metrics together with the consensus matrix patterns.

### 2.4 Feature selection

Effective feature selection not only improves classification performance but also reduces computational costs. Based on the structure of algorithmic models, feature selection methods are typically categorized into three types called filter, wrapper, and embedded (21). Filter methods rank features by calculating their importance and select a top proportion of features as the final feature subset for subsequent model training. Wrapper methods integrate the feature selection process with model training, using the output of the learning model as the evaluation criterion for features. These methods are often combined with classification algorithms such as Support Vector Machine (SVM) or Random Forest (RF) to improve classification accuracy and efficiency (22). Embedded methods are similar to wrapper methods, but feature selection is automatically performed during model training, typically relying on internal feature contribution metrics of the model (23).

As one of the wrapper methods, recursive Feature Elimination (RFE) employs a recursive process to eliminate less important features, thereby generating a series of candidate subsets from the complete feature space. It was introduced by Guyon et al. in 2002 and widely used in cancer classification (24). Compared to standard RFE, RFE with cross-validation (RFECV) incorporates cross-validation mechanisms, significantly enhancing the robustness of the feature selection process (25). Our work employed RFECV combined with a multi-model voting strategy to identify stable and predictive features for the classification of novel GBM subtypes. Integrating multiple feature selection algorithms is expected to enhance performance while improving the stability of feature selection (26). Therefore, we utilized three machine learning algorithms of Random Forest (RF), Support Vector Machine (SVM) and Extreme Gradient Boosting (XGBoost) as feature importance evaluators.

For the 347 patient samples in GEO database, we randomly divided them into a training set and an independent test set according to the ratio of 7:3. Accordingly, 242 samples were used for model construction with five-fold cross-validation, whereas the remaining 105 samples were reserved as an independent test set to evaluate the predictive performance of the model. Here, RFECV was performed iteratively on the training set using five-fold stratified cross-validation, with classification accuracy serving as the performance metric. During each iteration, RFECV ranked features based on the model’s performance on the validation set and recorded the top-ranked features. To enhance the stability of feature selection, a voting strategy was adopted to integrate the results from different models. The frequency of each feature being selected as the top feature across multiple iterations and models was statistically analyzed. Ultimately, features that were frequently selected across the three models were considered as key predictive variables for distinguishing novel GBM subtypes.

### 2.5 Model construction and evaluation

We developed a machine learning prediction model based on methylation differences in ERS-related genes among the novel GBM subtypes, using RF as the classifier. RF is an ensemble learning method proposed by Breiman, which performs classification, regression, and other tasks by integrating multiple decision trees (27). Its advantages include high classification accuracy, suitability for high-dimensional data and the ability to evaluate feature importance (28). Before model construction, hyperparameter optimization was conducted using GridSearchCV from the Python scikit-learn library to ensure optimal model performance.

Our study employed cross-validation (CV) and Receiver Operating Characteristic (ROC) curve analysis for performance assessment. CV is particularly useful for small datasets, helping to prevent overfitting caused by poor data splitting (29). K-fold CV splits a dataset into K equal subsets. In each iteration, one subset is used as the validation fold, while the remaining ones are used for training.

The ROC curve is a widely used graphical tool for evaluating classification performance in machine learning. Unlike precision and recall, which are calculated at a specific classification threshold, the ROC curve illustrates model performance across a range of thresholds by plotting the True Positive Rate (TPR) against the False Positive Rate (FPR). The Area Under the Curve (AUC) quantifies the discriminative ability of the classifier, with higher values indicating better classification performance (30). An AUC of 0.5 indicates random guessing, while a value closer to 1 reflects better classification performance.

All final models were constructed using RF, and their predictive performance was primarily evaluated using four metrics, including accuracy (ACC), precision, recall, and F1 score. The corresponding equations for these evaluation metrics are shown below:


$$ACC=\frac{TP+TN}{TP+FP+TN+FN}$$

$$Precision=\frac{TP}{TP+FP}$$

$$Recall=\frac{TP}{TP+FN}$$

$$F1=\frac{2*Precision*Recall}{Precision+Recall}$$

Where TP, FP, TN and FN are the true positive, false positive, true negative and false negative, respectively.

### 2.6 Differential analysis

The TCGA-GBM dataset served as an external validation set for the classification model. To evaluate the validity of the novel molecular subtypes, a comprehensive analysis was performed, including survival differences, multiple immune features (immune cell infiltration, immune score, stromal score, immune checkpoint gene expression and tumor purity), and TMB.

Tumor tissues are highly complex, consisting of not only tumor cells but also various cells associated with the tumor microenvironment (TME). Studies have shown that immune cells play crucial roles in tumor growth, invasion, and metastasis, whereas stromal cells are involved in disease progression and drug resistance (30, 31). Therefore, this study employed the ESTIMATE algorithm to calculate immune scores, stromal scores, and tumor purity in GBM patients, aiming to assess the immune microenvironment characteristics of different subtypes.

We calculated the TIS score (T-cell Inflamed Score) to further evaluate the infiltration of T-cells. This score is based on the expression levels of T-cell subset-related gene sets (**Supplementary Table 2**) proposed by Şenbabaoğlu et al. (32), which include genes associated with CD8^+^ T cells, T helper cells and regulatory T cells. Antigen processing and presentation mechanism (APM) is a critical process in the immune system, ensuring the recognition and attack of invading pathogens and it includes peptide generation and trimming, peptide transport, assembly of the MHC class I loading complex and antigen presentation. These processes collectively influence the tumor’s ability to present antigens to immune cells, which is a fundamental determinant of immune response (32, 33). The APM score quantifies the expression levels of related genes (**Supplementary Table 3**), with research demonstrating its significant correlations with tumor grade, immune microenvironment characteristics and response to immunotherapy (34). To quantify cytolytic activity, the CYT score was calculated using the established method by Takahashi et al. (35). The calculation involves the geometric mean of the expression levels of two genes, GZMA and PRF1 (**Supplementary Table 4**). These genes are markers of cytotoxic T lymphocyte activity and are essential for inducing apoptosis in target cells, aiding in the clearance of infected or abnormal cells. Finally, our study calculated gene expression levels of exhausted CD8^+^ T cells as GET score. The score is derived from a gene set containing 21 genes (**Supplementary Table 5**) that show a positive correlation with PD-1 expression levels, reflecting the immunesuppression characteristics of different GBM subtypes (36). For APM, CYT, GET and TIS, matched TCGA gene expression data were converted from FPKM to TPM, and sample-level scores were calculated using the gsva() function in the GSVA R package with method = “ssgsea”.

TMB represents the number of somatic mutations in the coding regions of tumor cells, including single-base substitutions, insertions, and deletions. TMB reflects the mutation level of tumors and has been shown to correlate closely with the production of tumor neoantigens and the clinical efficacy of immunotherapy. Numerous studies have demonstrated a significant association between high TMB values and the effectiveness of immune checkpoint inhibitors (ICIs), although it may also indicate a higher tumor burden and poorer prognosis (37). We used the R package “maftools” to calculate the TMB levels for all GBM patients (38).

### 2.7 Cmap analysis

Differential expression analysis between Subtype 2 and other three subtypes was conducted on the TCGA mRNA expression data using the DESeq2 package. Genes with.log_2_FC| ≥ 1 and adjusted P value ≤ 0.01 were considered significantly differentially expressed, yielding 506 up-regulated and 150 down-regulated genes. For CMap analysis, the top 150 up-regulated genes combined with 150–150 down-regulated ones were selected as an exploratory transcriptional signature to be uploaded to the CLUE platform (https://clue.io) for candidate compound prediction. Connectivity scores ranging from -100 to 100 were used to evaluate the association between the query signature and compound-induced expression profiles. Negative scores suggest that a compound may reverse the query signature, whereas positive scores indicate a concordant transcriptional effect. Accordingly, compounds with strongly negative connectivity scores were considered preliminary candidate agents for further investigation (39, 40).

## 3 Results

### 3.1 ERS-related methylation profile construction for GBM patients

Initially, 1,380 ERS-related genes were mapped to the annotation file of the GEO methylation dataset to extract corresponding methylation site information. Ultimately, 25,518 ERS-related methylation sites were obtained, serving as the feature set for subsequent GBM subtype identification. Then, the standard deviation (SD) of each CpG site across all samples was calculated, and the distribution of SD values is shown in **Supplementary Figure 2**. Based on this distribution, a threshold of SD>0.2 was applied to identify probes with substantial variability among samples. Using this criterion, 925 highly variable methylation probes, accounting for 3.62% of the 25,518 ERS-related probes, were retained for subsequent analysis (**Supplementary Table 6**).

### 3.2 Unsupervised clustering by NMF

**Figure 2:**
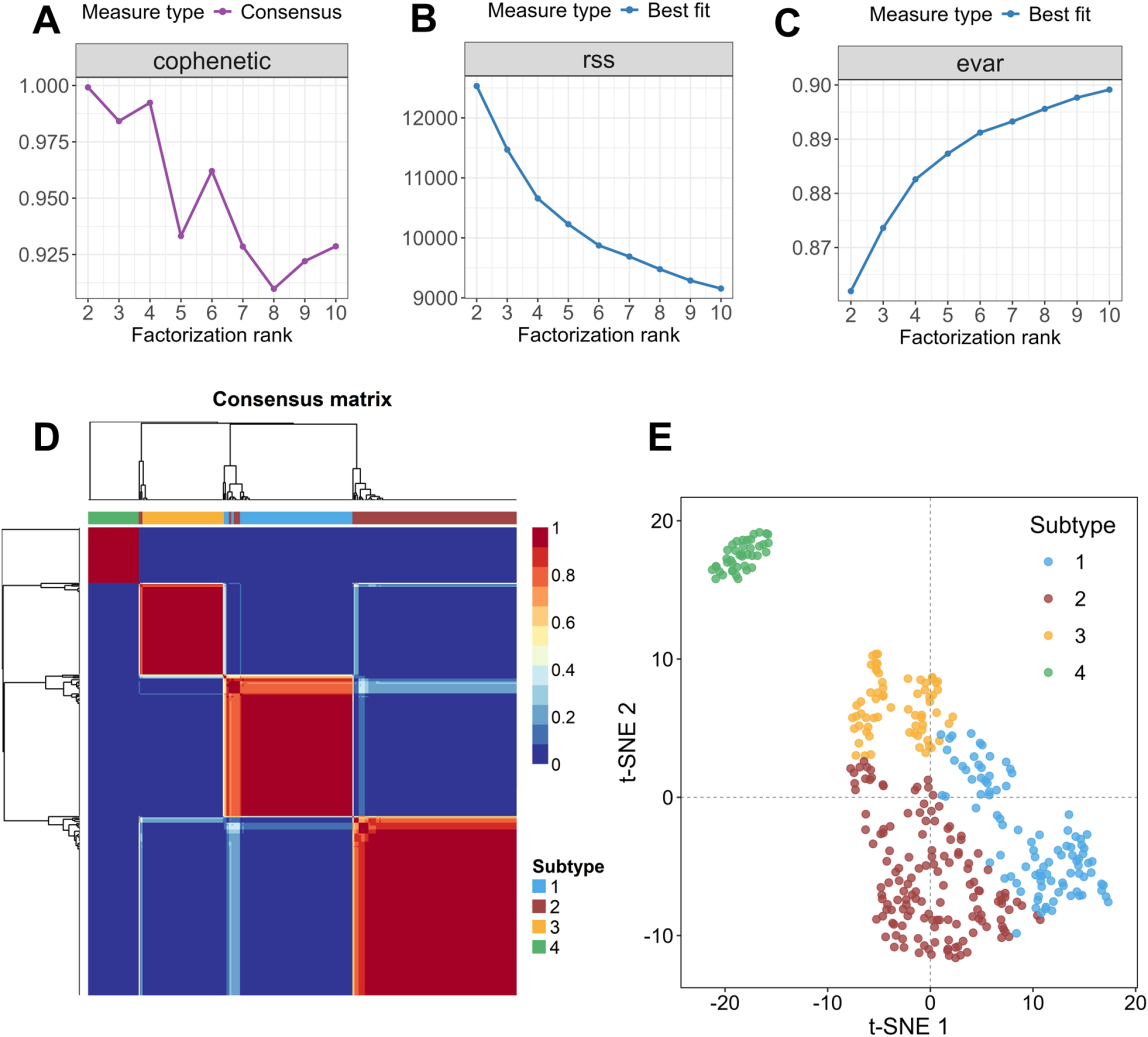
**(A-C)** Variations in cophenetic correlation, rss and evar across different factorization rank values. The number of clusters was determined based on the point preceding the steepest decline. **(D)** Consensus matrix shows that GBM patients are clearly classified into four subtypes, named as Subtype 1, 2, 3 and 4, respectively. **(E)** T-SNE visualization of four molecular subtypes.

For NMF clustering, the cophenetic correlation coefficient was first used to evaluate the clustering stability at various factorization ranks. As shown in **Figure 2A**, rank = 4 was identified as the optimal number of clusters according to the inflection point preceding the largest decline in the cophenetic correlation coefficient. At rank = 4, the cophenetic correlation coefficient remained relatively high, supporting the robustness of the clustering result. In addition, the residual sum of squares (RSS) and explained variance (EVAR) were jointly assessed (**Figures 2B, C**). The RSS decreased sharply from rank 2 to rank 4, indicating substantial improvement in model fit during this range. The slow decline in RSS at rank 4 suggests that further increases in rank would introduce minimal additional benefit while increasing the risk of overfitting. Meanwhile, EVAR increased steadily with rank and reached a relatively high value of 0.8826 at rank 4. Both evidences supported the selection of four as the optimal number of subtypes, ensuring model robustness and clear subtype separation.

In order to assess the rationale for using SD>0.2 for probe variability, we further performed sensitivity analyses using several SD thresholds of 0.15, 0.18, 0.20, 0.22 and 0.25, respectively. Across these thresholds, repeated NMF analyses consistently identified rank=4 as the optimal factorization rank (**Supplementary Figure 3**), indicating that the four-subtype solution is stable over a reasonable range of cutoff values. Under the adopted four-cluster solution, clustering stability remains high across the tested thresholds, with consistently favorable cophenetic correlation, silhouette consensus and dispersion metrics (**Supplementary Table 7**). Although the results obtained with SD>0.22 are close to those by SD> 0.20, the explained variance decreases from 0.883 to 0.864, suggesting reduced retention of methylation information under a more stringent filter. These findings support SD> 0.20 as a reasonable and balanced threshold for probe selection in the present study.

A consensus matrix was then generated to further assess clustering robustness (**Figure 2D**). The consensus matrix exhibited four well-defined diagonal blocks with consensus values close to 1, indicating that GBM samples were consistently assigned to four distinct subtypes. As depicted in **Figure 2E**, 347 GBM samples in GEO database were ultimately stratified into four subtypes based on the differential methylation patterns of ERS-related genes, designated as Subtype 1, Subtype 2, Subtype 3 and Subtype 4, with corresponding sample sizes of 97, 143, 66 and 41, respectively.

### 3.3 In silico prediction and characterization of the new subtypes in the GEO cohort

**Figure 3:**
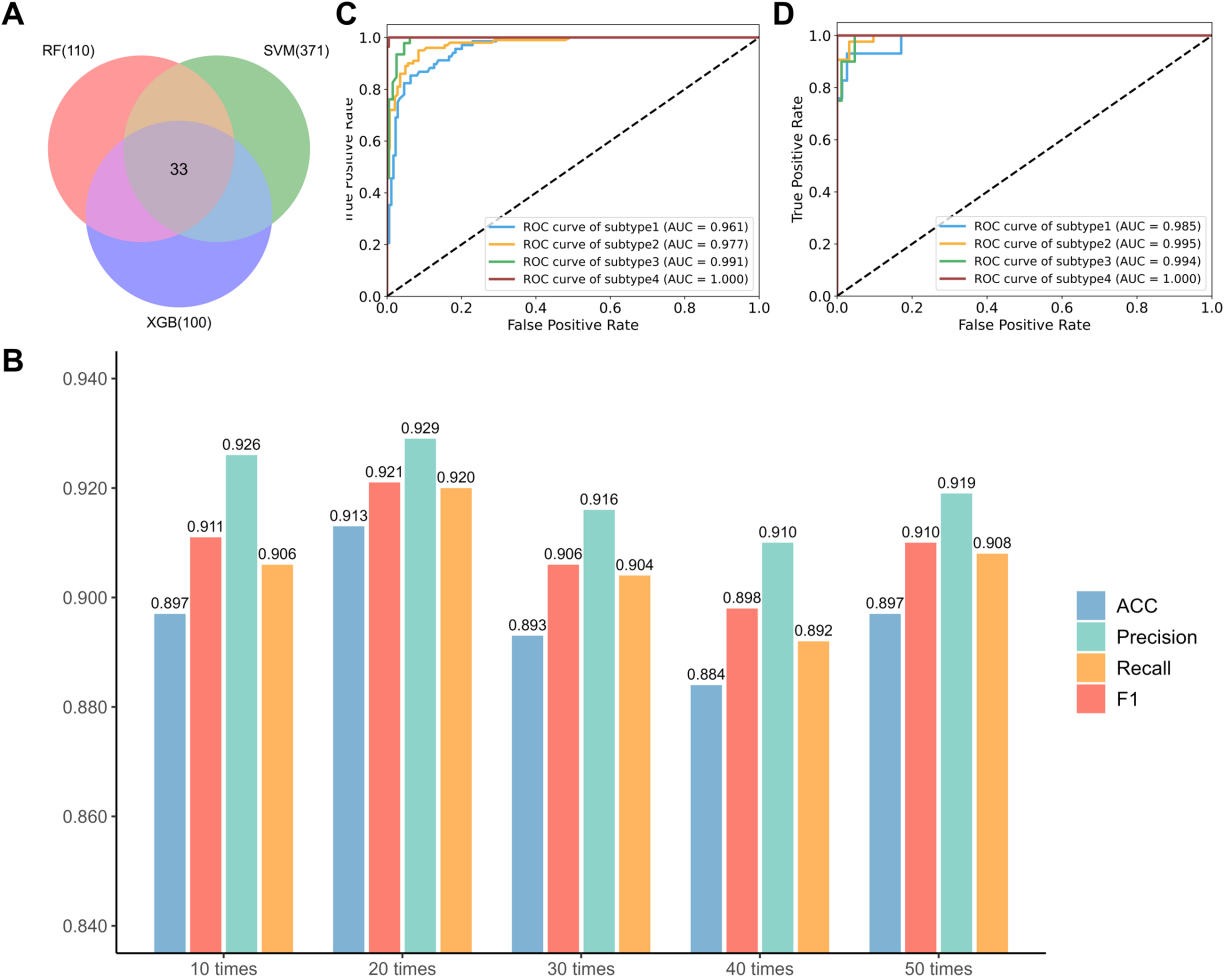
**(A)** Visualization of intersecting CpG probes. **(B)** Performance comparison of RF models based on different iterations. **(C, D)** ROC validation of classification models on the training and independent test sets, respectively.

Furthermore, a classification model was established for distinguishing the four new subtypes by RF. Considering the imbalance between the limited sample size and the large number of features, RFECV was performed for feature selection. Here, RFECV experiments were performed for 50 runs and the results show that with the 20^th^ run, the RF, SVM and XGB estimators yield 110, 371 and 100 CpG site features respectively and the RF model on the 33 overlapped features gives the best performance (**Figures 3A, B**). The optimal 33 features are listed in **Supplementary Table 8**. We can see that the final RF model achieves high AUC values over 0.96 for all subtypes in **Figure 3C** on the training set by 5-fold CV and the detailed prediction results are listed in **Table 1**. Then the model was further tested on the 105 independent test set. **Table 2** shows that the overall ACC is as high as 0.924 and the AUC values for the four subtypes are higher than 0.98 in **Figure 3D**, indicating good predictive performance of the final model in the independent test set. Consistent with these results, the overall classification performance remained stable in both the training and test sets, and the corresponding 95% confidence intervals and confusion matrices are provided in **Supplementary Tables 9**, **10**; **Supplementary Figure 4**, respectively.

**Table 1:** Classification performance of the model for identifying four subtypes based on 33 features using 5-fold CV on the training set.

Subtypes	ACC	Precision	Recall	F1
Subtype 1	–	0.889	0.824	0.855
Subtype 2	–	0.922	0.940	0.931
Subtype 3	–	0.880	0.957	0.917
Subtype 4	–	1.000	0.964	0.982
Overall	0.913 ± 0.040	0.929 ± 0.036	0.920 ± 0.038	0.921 ± 0.039

**Table 2:** Classification performance of the model for identifying four subtypes based on 33 features on the independent test set.

Subtypes	ACC	Precision	Recall	F1
Subtype 1	–	0.929	0.897	0.912
Subtype 2	–	0.911	0.953	0.932
Subtype 3	–	0.944	0.850	0.895
Subtype 4	–	0.929	1.000	0.963
Overall	0.924	0.928	0.925	0.925

Here, we also compared the four subtypes with established GBM classification systems in the GEO cohort, including Capper and Ceccarelli frameworks. The corresponding subtype annotations were obtained from previously published sources and matched to the GEO samples based on sample identifiers. The comparative heatmaps are presented in **Supplementary Figure 5**. Cross-tabulation analysis shows the existing of relationships between the ERS-related methylation subtypes and both classification systems, but yields much more difference. In Capper framework, Subtype 4 shows the closest correspondence with GBM_G34, whereas Subtype 1 is mainly associated with GBM_RTK_I and GBM_RTK_II, Subtype 2 with GBM_MES, GBM_MYCN and GBM_RTK_II, and Subtype 3 with GBM_MID, GBM_RTK_I and GBM_RTK_III;. In the Ceccarelli framework, Subtype 1 is concentrated in LGm4 and LGm5, Subtype 2 is across LGm4, LGm5 and LGm6, and Subtypes 3 and 4 are more frequently associated with LGm5 and LGm6. These findings suggest that the proposed ERS-related methylation subtypes are related to the established GBM classification frameworks, while also preserving distinct ERS-associated stratification patterns. Complete contingency tables and residual statistics are provided in **Supplementary Tables 11**, **12**.

Further, to improve the interpretability of the classifier, we examined the relative importance of the 33 selected CpG probes in the final model. The contributions of individual CpG probes to subtype classification have been shown in **Supplementary Figure 6**. Among them, cg25285646 shows the highest importance, followed by cg19366147, cg06902379 and cg13634501, etc., suggesting that the classification performance of the model is driven by a limited subset of highly informative ERS-associated methylation loci rather than by all probes contributing equally. To further characterize the biological context of these key features, the top 10 CpG probes were annotated according to their mapped genes, genomic regions and CpG island context, which are summarized in **Supplementary Table 13**. Notably, two highly ranked probes, cg13634501 and cg13798970, are both mapped to ANXA2, and locate in the 5’UTR and TSS200 regions, respectively, indicating that this locus lies within promoter-proximal regulatory regions and may therefore be relevant to transcriptional control. ANXA2 has been widely reported to be dysregulated in multiple cancers, and studies in glioma have linked its upregulation to invasion, angiogenesis, proliferation and aggressive tumor behavior (41). In addition, other highly ranked probes were mapped to genes with potential relevance to tumor biology, including MICA, PRKDC, HSPA2 and RASGRF2. MICA encodes a stress-inducible ligand of NKG2D that participates in antitumor immune recognition, and altered MICA/NKG2D signaling or MICA shedding has been associated with tumor immune escape in multiple cancer settings (42). PRKDC encodes the catalytic subunit of DNA dependent protein kinase and plays a central role in DNA double strand break repair, with abnormal activation being associated with genomic instability and therapy resistance (43). HSPA2, a member of the heat shock protein family, is involved in protein homeostasis and cellular stress responses. Although its role in cancer appears to be context dependent, previous studies have suggested associations with malignant phenotypes or prognosis in several tumor types (44). RASGRF2 encodes a Ras guanine nucleotide exchange factor and has been implicated in Ras-related signaling and migration or invasion-associated processes in certain tumor contexts (45).

Overall, these findings indicate that the methylation signature identified by the classifier is not randomly distributed across the genome, but is concentrated in ERS-related candidate genes and functionally relevant methylation regions. Although these results do not constitute direct functional validation, they provide additional biological context for the classifier and support that the selected CpG signature is associated with a biologically structured ERS-related methylation pattern rather than merely reflecting nonspecific heterogeneity.

### 3.4 Application of the model on TCGA data

**Figure 4:**
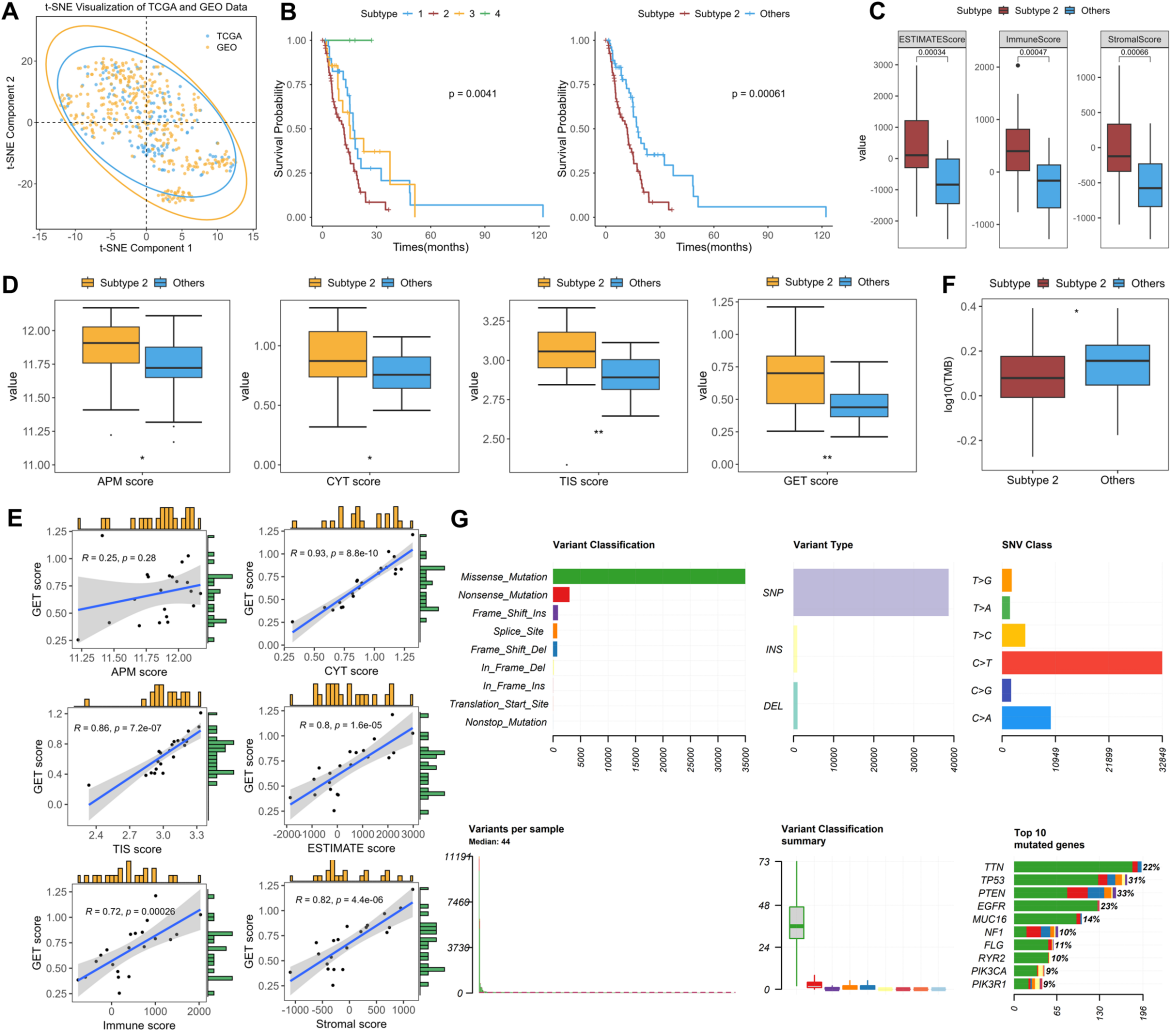
**(A)** T-SNE visualization of data distributions between TCGA and GEO cohorts. **(B)** Kaplan–Meier analysis of overall survival among the four subtypes (n = 123; Subtype 1, n = 30; Subtype 2, n = 69; Subtype 3, n = 21; Subtype 4, n = 3), showing significantly poorer survival in Subtype 2. A corresponding KM plot with the number-at-risk table is provided in **Supplementary Figure 7**. **(C, D)** Box plots for exploring the differences of APM, CYT, GET, TIS, immune and stromal scores, and tumor purity between Subtype 2 and other three subtypes (Subtype 2, n = 21; Others, n = 27). *p<0.05; **p<0.01. Exact p-values for the significant comparisons were as follows: APM, p = 0.0409; CYT, p = 0.0266; TIS, p = 0.0009; GET, p = 0.0014. **(E)** The correlations between GET score and the APM, CYT, TIS, immune and stromal scores, and tumor purity respectively, in Subtype 2 by Pearson’s correlation analysis. **(F)** Box plots of TMB score differences between Subtype 2 and other three subtypes (Subtype 2, n = 67; Others, n = 51; p = 0.0461). **(G)** Summary of mutation profiles.

As shown in **Figure 4A**, the TCGA-GBM methylation data exhibited a distribution similar to that of the GEO cohort, suggesting that the classification framework developed from the GEO dataset could be extended to the TCGA cohort. CpG probes were matched between the GEO and TCGA datasets by probe identifier, as both datasets were generated on the Illumina HumanMethylation450 platform. Among the 33 CpG probes included in the classifier, 25 are directly available in the processed TCGA dataset, whereas the remaining 8 probes are missing, so their corresponding β values were imputed using the mean values estimated from the GEO training cohort. Based on this aligned feature set, the constructed RF model was applied to 126 GBM samples from TCGA and successfully classified them into four subtypes (**Supplementary Table 14**).

To further evaluate the validity of the four TCGA subtypes, differences in clinicopathological, immune, and molecular features were compared across the four subtypes. Kaplan–Meier (KM) survival analysis was then performed to assess the association between the new molecular subtypes and OS in patients with TCGA-GBM. As shown in **Figure 4B**, OS differed significantly among the four subtypes (P = 0.0041). Further pairwise log-rank analyses with Benjamini–Hochberg correction shows that Subtype 2 exhibits significantly worse survival than the other three subtypes, whereas no significant survival differences are observed among Subtypes 1, 3 and 4. The detailed results are provided in **Supplementary Figure 7**; **Supplementary Table 15**. In addition, when Subtype 2 was compared with all other subtypes combined, a highly significant survival difference is still observed (log-rank test, P = 0.00061). Consistently, univariable Cox regression indicates that Subtype 2 is associated with significantly worse OS, with a hazard ratio of 2.236 (95% CI, 1.393 to 3.587, P = 0.00085), further supporting its unfavorable prognosis.

Immune feature analysis shows that Subtype 2 exhibits significantly higher ESTIMATE, immune, and stromal scores than the other subtypes (**Figure 4C**), suggesting a microenvironment with stronger immune and stromal involvement in this subtype. Consistent with the ESTIMATE framework, the higher ESTIMATE score also indicates relatively lower inferred tumor purity in Subtype 2. These findings suggest that immune related components may contribute substantially to the TME of Subtype 2. We compared the distribution of GET scores between Subtype 2 and other three subtypes to quantitatively assess the state of exhausted CD8^+^ T cells. Patients in Subtype 2 had significantly higher GET scores than those in the other subtypes, indicating a relatively stronger exhaustion related immune signature in this group (**Figure 4D**). Therefore, we further evaluated the relationships between GET scores and APM scores, CYT scores, TIS scores, immune scores, stromal scores and tumor purity within Subtype 2 samples. **Figure 4E** shows that GET scores were positively correlated with CYT scores, TIS scores, immune scores, stromal scores, and ESTIMATE scores within Subtype 2. Consistent with the ESTIMATE framework, this pattern also suggests an inverse relationship between GET scores and inferred tumor purity. CYT scores reflect cytolytic activity and have also been reported to be associated with T-cell exhaustion and inflammatory immune states in TME (46). Meanwhile, TIS comprises 18 genes that have been reported to correlate with clinical responses to immune checkpoint inhibitors and to reflect the extent of immune cell infiltration in TME. Taken together, these findings suggest that there may be coordinated or cooperative relationships among T-cell exhaustion, antigen presentation, and cytolytic activity in Subtype 2, which may contribute to a relatively inflammatory TME (**Supplementary Table 16**). Finally, we analyzed the gene mutation characteristics of all samples (**Figures 4F, G**). Missense mutations represents the dominant category among all mutation types, whereas nonsense and frameshift mutations are detected at lower frequencies, suggesting that many of these alterations may have functional consequences at the protein level. The majority of mutations are single nucleotide polymorphisms (SNPs), with C>T mutations being the most common, which is consistent with previously reported mutational patterns in GBM. The mutation burden analysis reveals considerable variability in the number of mutations across samples, with some samples exhibiting hypermutated phenotypes that may be potentially linked to DNA repair deficiencies. Among the frequently mutated genes, TP53 (31%), PTEN (33%), and EGFR (23%) were commonly altered, highlighting the involvement of pathways related to cell cycle regulation and PI3K/AKT signaling. However, Subtype 2 shows substantially lower TMB levels than other groups (p< 0.05), which warrants further investigation into the underlying biological mechanisms and their clinical implications.

### 3.5 Exploratory CMap analysis for candidate compound identification in Subtype 2

**Figure 5:**
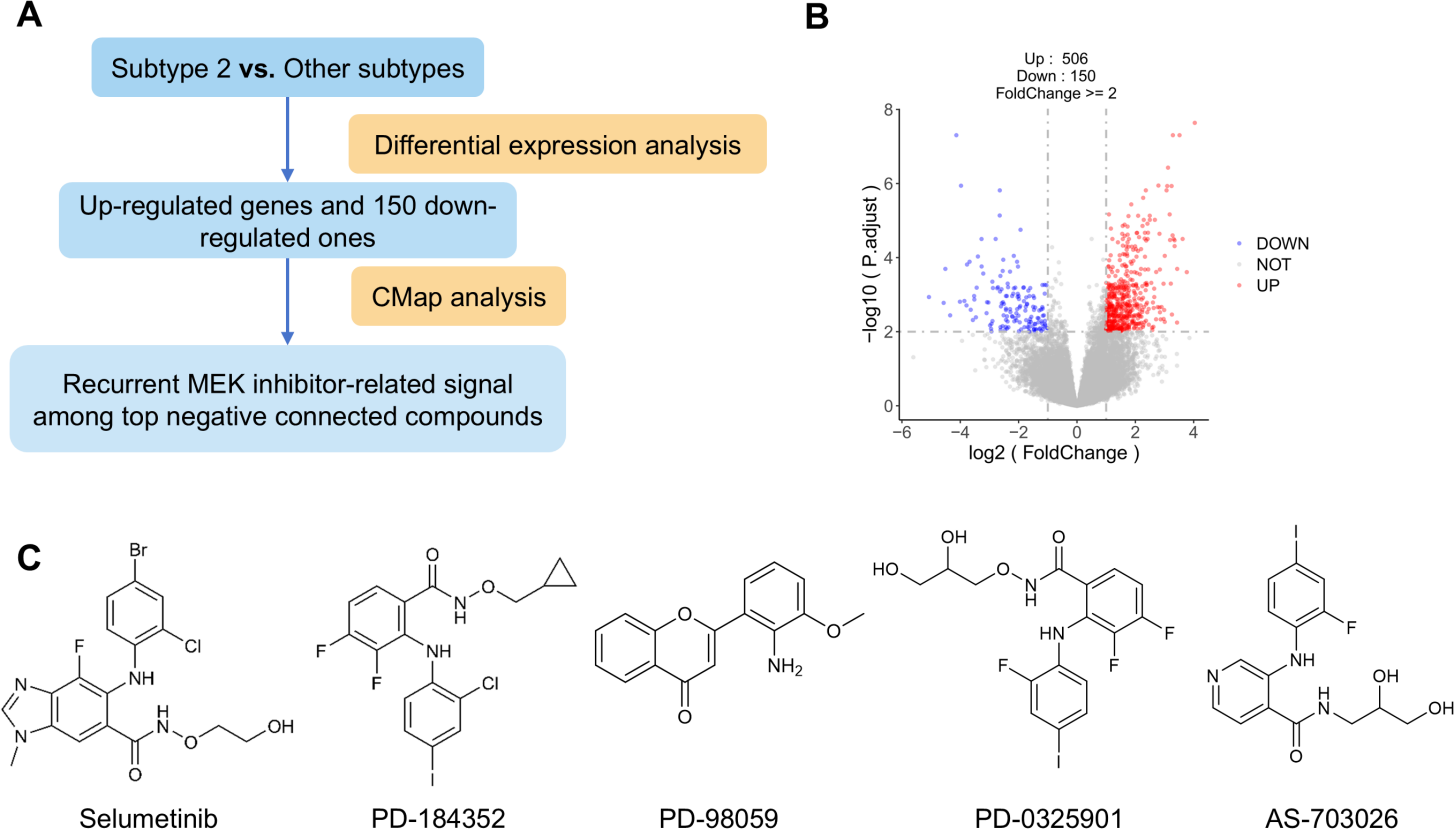
**(A)** Workflow of differential expression and Cmap analysis. **(B)** Volcano plot of differential gene expression for Subtype 2 versus other three subtypes. **(C)** Representative named MEK inhibitor-related compounds among the strongest negatively connected results are shown. The generic CMap annotation MEK inhibitor is listed in **Table 3** but is not shown here because it does not correspond to a uniquely defined chemical structure.

In our study, GBM are re-classified into four subtypes from ERS-related methylation patterns. Compared with Subtypes 1, 3 and 4, Subtype 2 is proved to be associated with shorter OS and higher immune heterogeneity. Given the high-risk characteristics, we next performed an exploratory CMap analysis using the top 150 up-regulated genes and 150 down-regulated ones in Subtype 2 as the query signature (**Figures 5A, B**). The analysis identified multiple strongly negatively connected compounds. Although the top negatively connected compounds were mechanistically heterogeneous overall, repeated occurrence of MEK inhibitor-related agents was observed among the strongest negative connections, including selumetinib, PD-184352, a generic MEK1/2 inhibitor annotation in CMap, PD-98059, PD-0325901 and AS-703026 (**Figure 5C**; **Table 3**). For example, selumetinib is a selective MEK1/2 inhibitor with an approved indication in NF1-associated plexiform neurofibromas, and preclinical work has also shown tumor reduction in an EGFR-positive glioblastoma xenograft model (47, 48). PD-98059 is a classical non-ATP-competitive inhibitor of the MEK pathway (49), whereas PD-0325901 has been reported to inhibit ERK phosphorylation and reduce dispersal and growth in glioblastoma models (50). Similarly, AS-703026 is a selective MEK1/2 inhibitor with reported anti-tumor activity in multiple myeloma and KRAS-mutant colorectal cancer models (51, 52). In addition, previous studies have suggested potential crosstalk between MEK/ERK signaling and ER stress-related responses, including ERN1-dependent modulation of sensitivity to MEK inhibition in KRAS-mutant cancers (53, 54). So these observations provide biological context for the recurrent appearance of MEK inhibitor-related agents among the strongest negatively connected compounds in our CMap analysis. However, the CMap results represent transcriptional reversal signals and do not in themselves establish subtype-specific therapeutic efficacy. Further mechanistic and experimental studies will therefore be needed to validate these candidate compounds.

**Table 3:** Representative negatively connected compounds identified by CMap analysis in Subtype 2, including recurrent MEK inhibitor-related agents.

Name	Score	Target	MOA
Selumetinib	-97.81	MAP2K1, MAP2K2	MEK inhibitor
PD-184352	-98.38	MAP2K1, MAP2K2, MAP3K1, MAP3K2	MEK inhibitor
MEK1-2-inhibitor	-98.77	MAP2K1, MAP2K2	MEK inhibitor
PD-98059	-99.37	MAP2K1, AKT1, CHEK1, GSK3B, LCK, MAP2K2, MAPK1, MAPK11, MAPK12, MAPK14, MAPK3, MAPK8, PRKCA, RAF1, ROCK1, RPS6KB1, SGK1	MEK inhibitor, MAP kinase inhibitor
PD-0325901	-99.47	MAP2K1, MAP2K2	MEK inhibitor, MAP kinase inhibitor, Protein kinase inhibitor
AS-703026	-99.61	MAP2K1, MAP2K2	MEK inhibitor

## 4 Discussion

As an extremely malignant intracranial tumor, the current standard treatment regimens for GBM include surgical resection, radiotherapy and temozolomide-based chemotherapy. However, the long-term clinical prognosis for patients remains unsatisfactory. Existing treatment methods often come with severe side effects. The high heterogeneity of GBM is a key factor affecting treatment efficacy. Traditional WHO grading cannot accurately distinguish patients with different clinical outcomes. In-depth analysis of the genomic characteristics of GBM patients helps to identify new prognostic biomarkers and clinical therapeutic targets, thereby promoting the development of personalized treatment strategies (55).

Studies have shown that the regulation of ER stress response involves multi-level epigenetic modifications, including DNA methylation and histone modifications. These epigenetic changes may affect the activation status of the ER stress pathway. For example, hypermethylation at specific CpG sites may inhibit the binding of transcription factors, thereby reducing gene expression (56). Additionally, differential expression of non-coding RNAs, especially microRNAs, has been shown to fine-tune the expression levels of ERS-related genes by targeting their mRNAs (57). In this context, our study investigated the associations of ERS-related DNA methylation features with GBM clinical phenotypes, molecular stratification, and prognosis, with the aim of clarifying whether ERS-associated epigenetic variation may contribute to biological heterogeneity in GBM.

Against this background, we performed NMF clustering based on ERS-related methylation patterns and identified four ERS-associated methylation subtypes in GBM. These results suggest that ERS-related methylation patterns may serve as candidate biomarkers for GBM stratification and characterization. We further applied machine learning methods to construct a classification model based on ERS-related methylation features. High-dimensional features often contain redundant or irrelevant information, which can result in model overfitting and diminished predictive performance. To mitigate these issues, we implemented cross-validated recursive feature elimination (RFECV) for feature selection, incorporating a multi-model voting strategy to identify a final subset of 33 methylation sites. The RF model achieved an overall accuracy of 92.4%, supporting the predictive utility of the selected methylation probes. When applied to the TCGA-GBM cohort, the classifier assigned the 126 primary tumor samples into the four ERS-associated methylation subtypes.

Studies have shown that ERS plays an important role in TME (58). The main components of TME include tumor cells, immune cells, stromal cells, the vascular system and the extracellular matrix (ECM) (59). Its complexity determines tumor growth, progression and resistance. The proportion of tumor cells in the TME reflects tumor purity, with lower purity in GBM cases being associated with a more pronounced malignancy, often leading to poor prognosis. Comparative analyses show that Subtype 2 is characterized by relatively higher APM, CYT and TIS scores, together with lower tumor purity. APM has been reported to reflect antigen presentation activity and has been linked to immunoregulatory features in the tumor microenvironment. A high CYT score is associated with high expression of the PD1/PD-L1 axis and leading to poor OS in GBM. In our cohort, Subtype 2 also showed higher ESTIMATE scores and lower tumor purity, together with poorer survival outcomes. Taken together, these findings suggest that Subtype 2 may represent a more immune-enriched yet clinically adverse microenvironment (60). However, whether this subtype would derive greater benefit from immunotherapy cannot be determined from the present data and requires further validation in immunotherapy-treated cohorts. Therefore, Subtype 2 appears to be associated with poorer prognosis and altered immune-related characteristics, but its potential sensitivity to immunotherapy remains speculative.

Compared with Subtypes 1, 3 and 4, Subtype 2 was associated with poorer OS and a more heterogeneous immune microenvironment. In view of these unfavorable characteristics, we further interpreted the CMap results derived from the top 150 upregulated genes in Subtype 2. Among the strongest negatively connected compounds, several MEK inhibitor related agents were recurrently identified. This recurrent signal is of potential interest because previous studies have suggested interplay between MEK/ERK signaling and ER stress related responses. For example, ER stress can be influenced by MAPK pathway activity, whereas MEK inhibition has also been linked to altered unfolded protein response programs in some tumor contexts (61, 62). In addition, some of these compounds have shown anti-tumor activity in glioblastoma or other cancer models. Taken together, these observations provide a plausible biological context for the repeated appearance of MEK inhibitor related compounds in the CMap analysis of Subtype 2. However, these results should be interpreted as exploratory, and the therapeutic relevance of these compounds in GBM, particularly in the ERS-associated subtype identified here, requires further experimental validation.

However limitations of this study should be acknowledged. As a retrospective analysis based on publicly available datasets, the findings require further validation in prospective cohorts. In addition, differences in platforms and preprocessing procedures between datasets may have affected comparability. The biological relevance of the identified ERS-related methylation subtypes was inferred mainly from computational analyses and still lacks direct wet-lab validation. Moreover, although tumor purity was evaluated, its potential confounding effects on subtype classification and downstream analyses cannot be fully excluded.

## 5 Conclusion

Overall, our study established a novel GBM classification framework based on ERS-related DNA methylation patterns and developed a machine learning model that accurately distinguished four subtypes. When this framework was applied to the TCGA cohort, Subtype 2 showed significantly poorer survival than the other subtypes. Further analyses suggested that this subtype was also associated with distinct tumor purity, TMB and immune microenvironment characteristics. These findings indicate that ERS-related methylation patterns may contribute to molecular heterogeneity in GBM and may be associated with differences in disease progression and the tumor microenvironment. In addition, CMap analysis identified several MEK inhibitors as preliminary candidate compounds for Subtype 2, although their therapeutic relevance remains to be validated experimentally. Collectively, this work provides an epigenetic perspective on the role of ERS in GBM and may support future studies aimed at refining molecular stratification and exploring subtype-related therapeutic strategies.

## Data Availability

The original contributions presented in the study are included in the article/**Supplementary Material**. The 33-CpG signature, model code, and analysis scripts used in this study are publicly available at GitHub (https://github.com/jyz30102/ERSR-GBM-Subtypes). Further inquiries can be directed to the corresponding authors.
