# Supplementary material for: Machine-learning assisted subclassification of glioblastoma by developing an endoplasmic reticulum stress-related methylation signature
Source: Front Oncol. 2026 Apr 22;16:1750334. doi: 10.3389/fonc.2026.1750334 (PMC13143586; doi:10.3389/fonc.2026.1750334)
Supplement: Supplementary file 1 [file DataSheet1.docx]

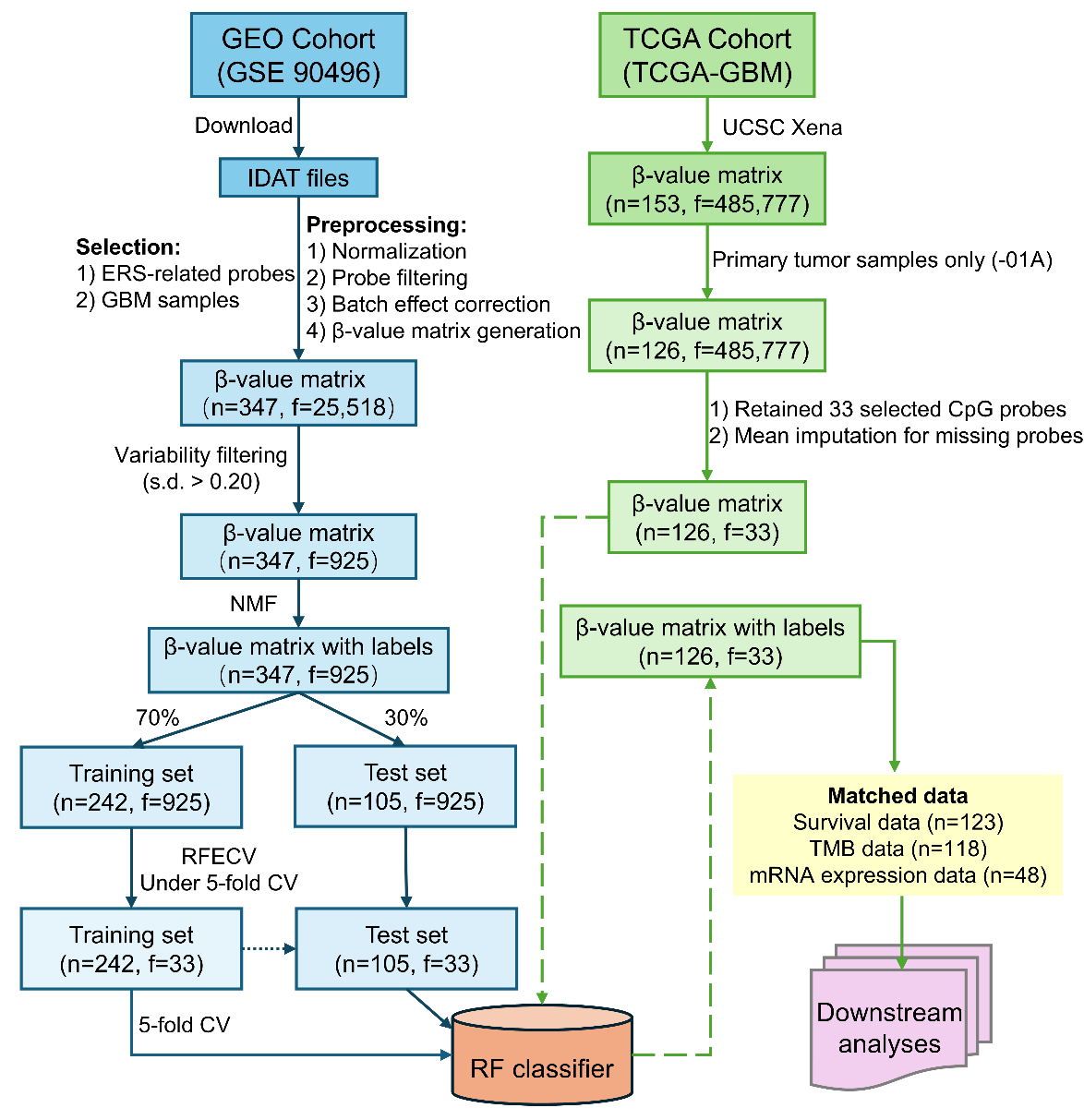


**Figure S1** | Schematic overview of cohort processing, subtype discovery, classifier development, and downstream analyses. Here, **n** denotes the number of samples and **f** denotes the number of features.


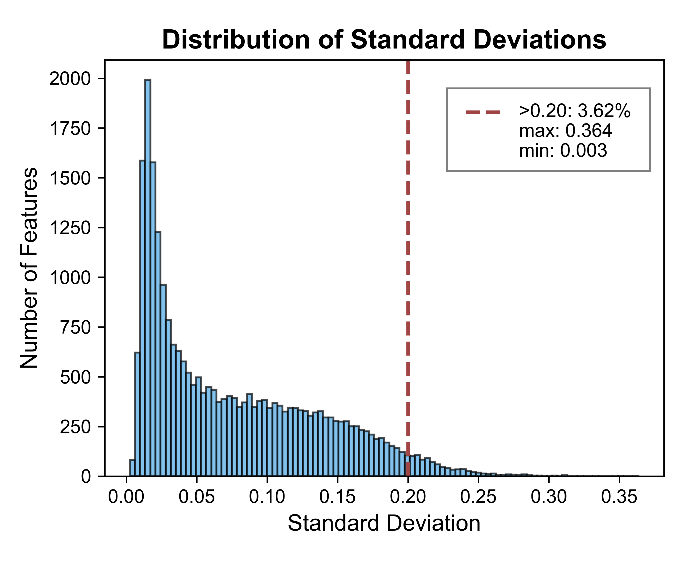


**Figure S2** | The distribution of standard deviations of CpG methylation β-values across all probes in the dataset.


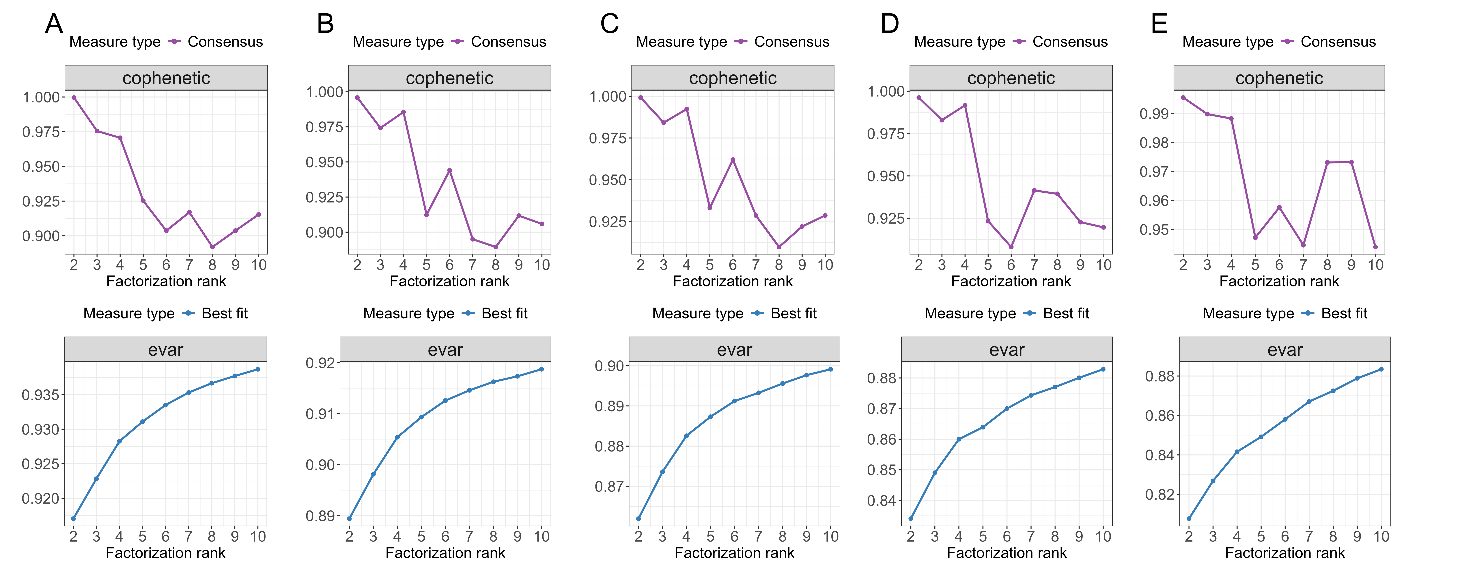


**Figure S3** | Trends of the cophenetic correlation coefficient and explained variance across ranks 2 to 10 under different s.d. thresholds. The choice of s.d. threshold did not affect the selection of rank 4 as the optimal number of NMF clusters.


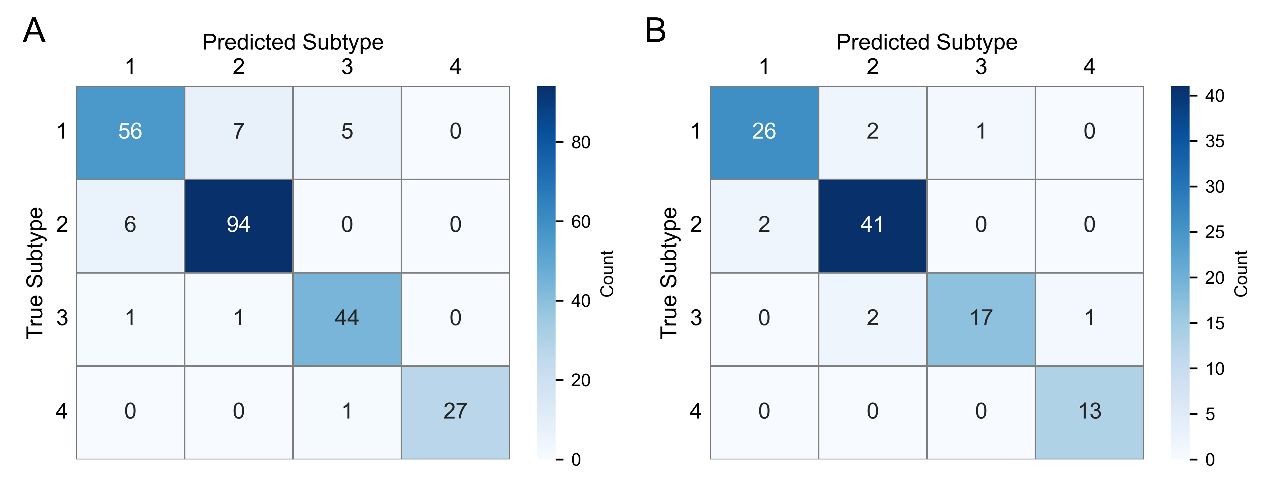


**Figure S4** | Confusion matix. (A) Cross-validation on training set. (B) On independent set.


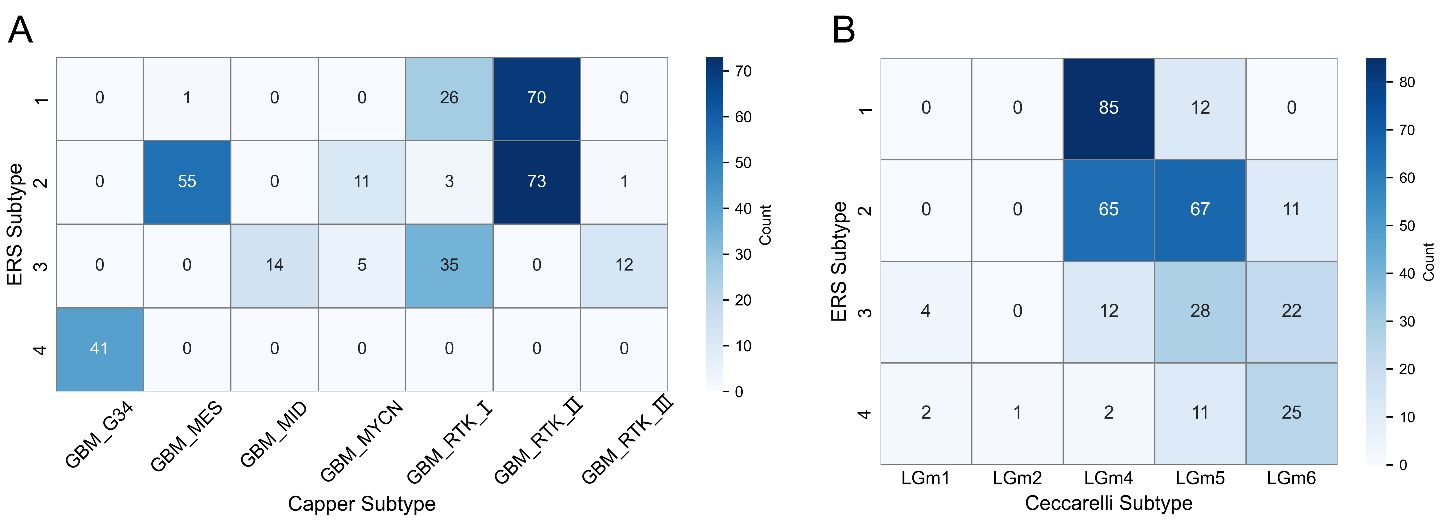


**Figure S5** | Difference heatmaps. (A) ERS-related methylation subtypes vs Ceccarelli classification. (B) ERS-related methylation subtypes vs Capper classification.


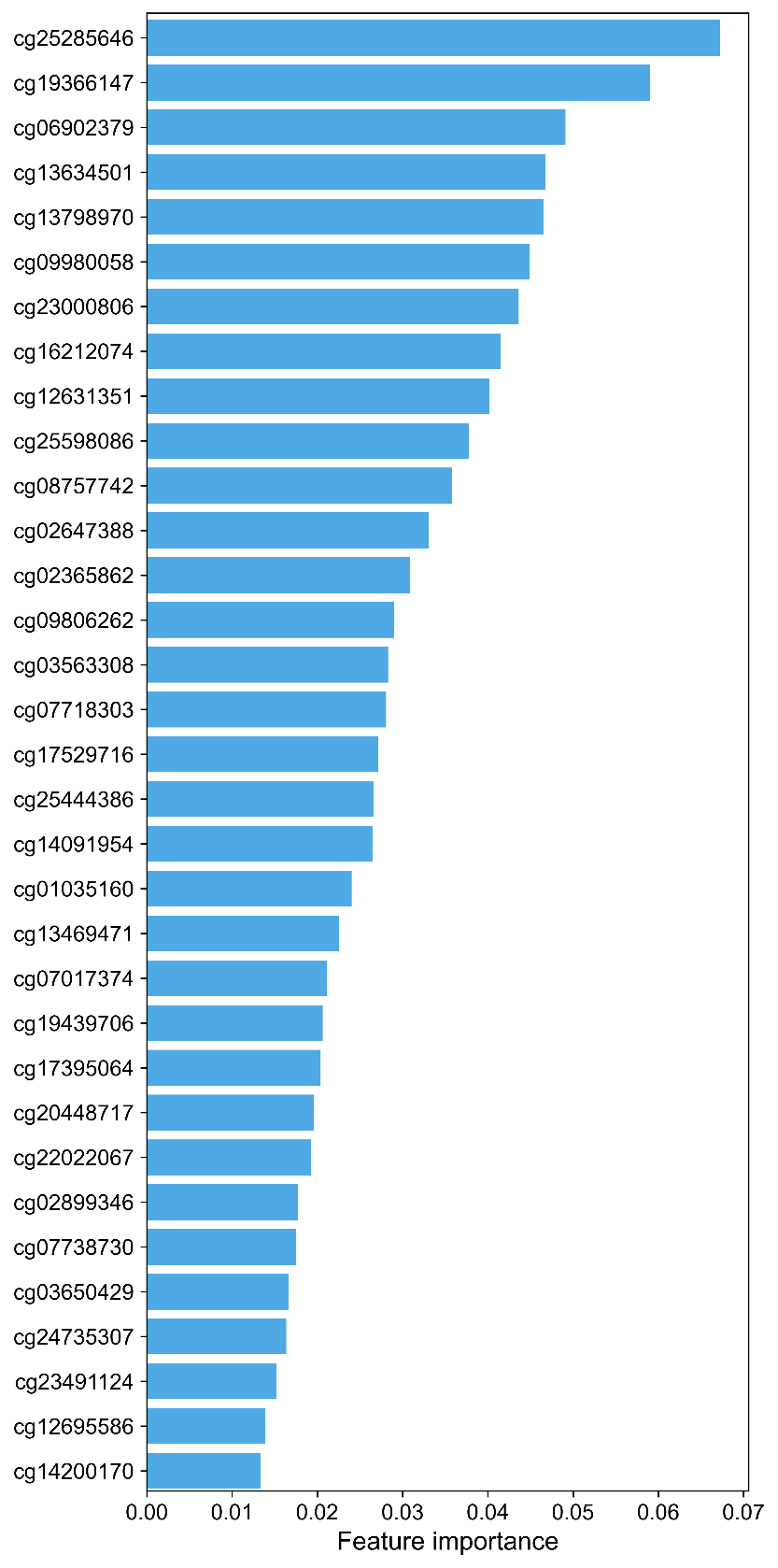


**Figure S6** | Top 10 important CpG probes in the RF classifier.


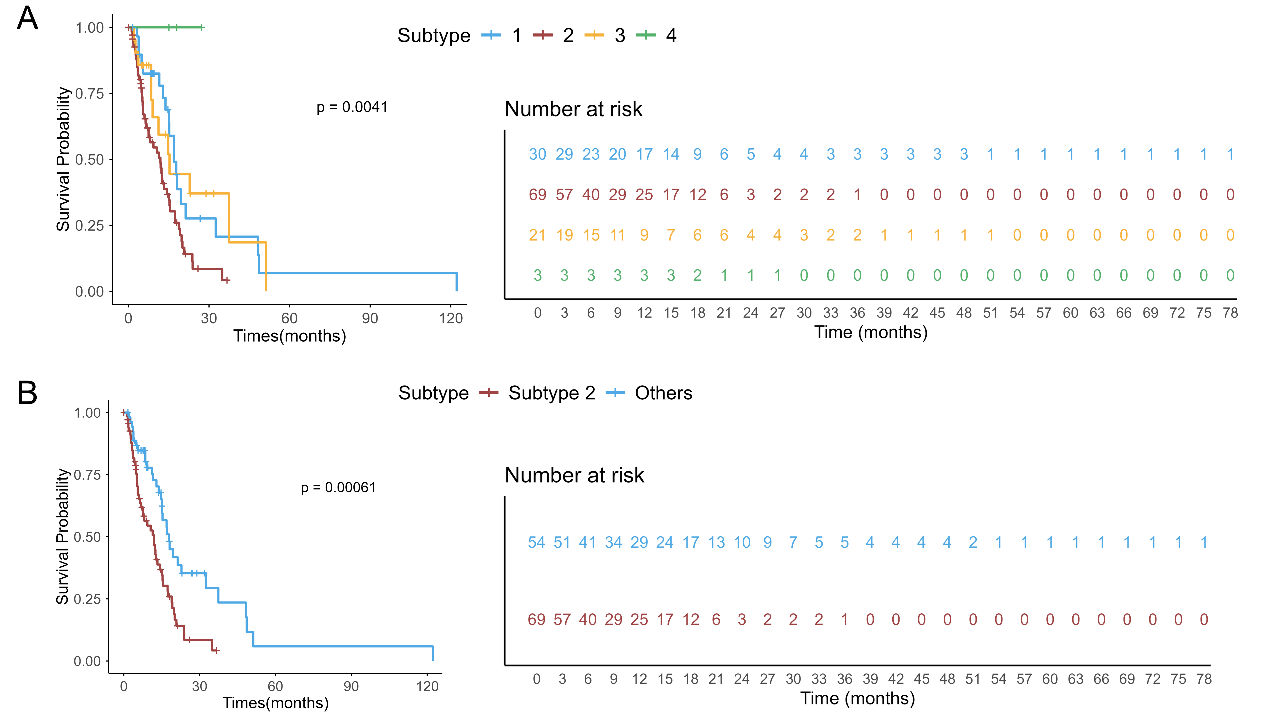


**Figure S7** | KM survival analyses with number-at-risk tables in the TCGA cohort. (A) Among four subtypes. (B) Between Subtype 2 and other three subtypes.

**Table S1** | ERS-related genes.

| 1 | RYR2 | 231 | SCARNA5 | 461 | SEC23IP | 691 | JPH1 | 921 | UGT1A9 | 1151 | CASR |
| --- | --- | --- | --- | --- | --- | --- | --- | --- | --- | --- | --- |
| 2 | HSPA5 | 232 | NFKB1 | 462 | DNAJB14 | 692 | SHISA5 | 922 | TRAP1 | 1152 | TLR3 |
| 3 | ERN1 | 233 | ESYT1 | 463 | UMOD | 693 | RPL5 | 923 | UGT1A4 | 1153 | CYP51A1 |
| 4 | TRDN | 234 | RRBP1 | 464 | RNF139 | 694 | SSR1 | 924 | PSMA7 | 1154 | CUL1 |
| 5 | ATP2A2 | 235 | ERGIC2 | 465 | DNAJB12 | 695 | GADD45A | 925 | MR1 | 1155 | LGALS1 |
| 6 | SERP1 | 236 | NOX4 | 466 | UBAC2 | 696 | YKT6 | 926 | PDIA5 | 1156 | PEMT |
| 7 | EIF2AK3 | 237 | ATL3 | 467 | HM13 | 697 | SAMD8 | 927 | STK25 | 1157 | CAMK2D |
| 8 | XBP1 | 238 | APOE | 468 | CCDC88B | 698 | SACM1L | 928 | ABCD1 | 1158 | ATF1 |
| 9 | CASQ2 | 239 | FAF2 | 469 | SCAMP5 | 699 | TGM2 | 929 | KPNA2 | 1159 | PLEC |
| 10 | ERAP1 | 240 | TOR1A | 470 | THBS1 | 700 | EBP | 930 | ROCK1 | 1160 | UBQLN4 |
| 11 | ATP2A3 | 241 | RPS27A | 471 | UGT1A6 | 701 | RHBDD1 | 931 | OPRM1 | 1161 | NPHS2 |
| 12 | ERO1A | 242 | RAB1A | 472 | CALM2 | 702 | MT-TP | 932 | USP19 | 1162 | SLC6A3 |
| 13 | ATP2A1 | 243 | CCL2 | 473 | TMTC3 | 703 | C2CD2L | 933 | SIRT6 | 1163 | UBXN2B |
| 14 | ATF6 | 244 | ORAI1 | 474 | IL1A | 704 | ZFYVE1 | 934 | RNF13 | 1164 | ELOVL4 |
| 15 | VCP | 245 | CACNA1S | 475 | AGER | 705 | CDH1 | 935 | GABRG2 | 1165 | NOX1 |
| 16 | ERP29 | 246 | XDH | 476 | ATR | 706 | TMEM41B | 936 | PRKDC | 1166 | HBB |
| 17 | SERP2 | 247 | PPIB | 477 | VIM | 707 | YWHAE | 937 | ECPAS | 1167 | PLA2G6 |
| 18 | HERPUD1 | 248 | TRAM1 | 478 | RNFT1 | 708 | GDF15 | 938 | UBE2D2 | 1168 | HSD17B2 |
| 19 | ERP44 | 249 | SERPINH1 | 479 | PRDX6 | 709 | KDR | 939 | BFAR | 1169 | CPT1C |
| 20 | ERAP2 | 250 | HSD17B10 | 480 | EMD | 710 | MTDH | 940 | TREX1 | 1170 | YWHAQ |
| 21 | DDIT3 | 251 | PON1 | 481 | STAT3 | 711 | CAMK2B | 941 | TMEM106C | 1171 | YOD1 |
| 22 | ERGIC1 | 252 | CISD2 | 482 | EMC1 | 712 | UFM1 | 942 | FANCD2 | 1172 | SERPINC1 |
| 23 | KDELR1 | 253 | GSK3B | 483 | SLC8A1 | 713 | RFT1 | 943 | SERPINE1 | 1173 | MATN3 |
| 24 | OS9 | 254 | SEC24B | 484 | ACE | 714 | DPM3 | 944 | VRK2 | 1174 | POMGNT2 |
| 25 | KDELR2 | 255 | FKBP14 | 485 | MAPK13 | 715 | FKBP10 | 945 | MMP2 | 1175 | TMEM67 |
| 26 | ATF4 | 256 | MAPK3 | 486 | MIR211 | 716 | MAP3K7 | 946 | SCAPER | 1176 | TUBB4A |
| 27 | ERN2 | 257 | UBE2J2 | 487 | SEC23B | 717 | CTSB | 947 | HSPA1L | 1177 | DDX1 |
| 28 | ERLEC1 | 258 | EGFR | 488 | IFNG | 718 | UBA5 | 948 | UGT1A8 | 1178 | AVP |
| 29 | TP53 | 259 | ASPH | 489 | MIR320A | 719 | KHSRP | 949 | MIR125A | 1179 | HMGCL |
| 30 | CALR | 260 | NQO1 | 490 | TOR1AIP2 | 720 | SEC11A | 950 | UCHL1 | 1180 | HADHB |
| 31 | ERO1B | 261 | HERPUD2 | 491 | AHCYL1 | 721 | ELAVL1 | 951 | BAD | 1181 | CCDC88A |
| 32 | KDELR3 | 262 | CANT1 | 492 | CD4 | 722 | BAG3 | 952 | RPS3 | 1182 | MEG8 |
| 33 | SEC16A | 263 | NOS2 | 493 | MAP1LC3A | 723 | SCFD1 | 953 | VANGL1 | 1183 | TMEM238L |
| 34 | SOD2-OT1 | 264 | CRHR1 | 494 | GJA1 | 724 | TXNRD1 | 954 | CYP2C19 | 1184 | EIF2B3 |
| 35 | HSP90B1 | 265 | TLR4 | 495 | KTN1 | 725 | HMOX2 | 955 | DNAJB2 | 1185 | PGRMC1 |
| 36 | BDNF-AS | 266 | RAB1B | 496 | CAMLG | 726 | NGLY1 | 956 | PITPNM1 | 1186 | GPAT3 |
| 37 | EIF2S1 | 267 | MAPK9 | 497 | TRIM13 | 727 | ASNS | 957 | ATP13A2 | 1187 | FKBP1B |
| 38 | ERP27 | 268 | STUB1 | 498 | PTEN | 728 | EMC6 | 958 | GLA | 1188 | PSENEN |
| 39 | RER1 | 269 | STIM2 | 499 | TRIB3 | 729 | MMGT1 | 959 | AQP8 | 1189 | SPCS3 |
| 40 | CANX | 270 | BACE1 | 500 | RHOA | 730 | CD36 | 960 | NACA | 1190 | COPB2 |
| 41 | LOC126806068 | 271 | GET4 | 501 | CASP12 | 731 | FUS | 961 | MAP3K20 | 1191 | SCN1A |
| 42 | ERMP1 | 272 | BAK1 | 502 | HLA-DRB1 | 732 | ZC3H12A | 962 | F7 | 1192 | UGT1A3 |
| 43 | MAPK8 | 273 | TAP1 | 503 | OXT | 733 | CDK5 | 963 | ATP2C1 | 1193 | CBY1 |
| 44 | PDIA3 | 274 | SEC63 | 504 | JKAMP | 734 | MSRB3 | 964 | AR | 1194 | PHB2 |
| 45 | CHERP | 275 | INSIG2 | 505 | GPX8 | 735 | PPP1CC | 965 | HADHA | 1195 | UBE2K |
| 46 | HMOX1 | 276 | TXNIP | 506 | ITPR3 | 736 | HACD3 | 966 | GCN1 | 1196 | TH |
| 47 | RYR1 | 277 | ATM | 507 | AGR2 | 737 | LINC01554 | 967 | TUSC3 | 1197 | HRAS |
| 48 | CPT2 | 278 | SEC62 | 508 | CAST | 738 | CAPRIN1 | 968 | ATP1A3 | 1198 | SGPL1 |
| 49 | BCL2 | 279 | PRKAA1 | 509 | MAP2K1 | 739 | CTNNB1 | 969 | POMT1 | 1199 | ACER1 |
| 50 | NFE2L2 | 280 | SRP54 | 510 | CDKN3 | 740 | GABARAP | 970 | UBXN2A | 1200 | SIRT2 |
| 51 | APP | 281 | POMC | 511 | TTR | 741 | F8 | 971 | KRT5 | 1201 | SELP |
| 52 | SYVN1 | 282 | RTN1 | 512 | SP1 | 742 | SI | 972 | MUC5AC | 1202 | RPS23 |
| 53 | P4HB | 283 | ARL6IP1 | 513 | MTTP | 743 | SET | 973 | TIAL1 | 1203 | ARSH |
| 54 | LINC01672 | 284 | GPR37 | 514 | P3H1 | 744 | ATG14 | 974 | KCNE2 | 1204 | DCSTAMP |
| 55 | SOD1 | 285 | ESYT2 | 515 | HSP90AB1 | 745 | OSBPL5 | 975 | MAOB | 1205 | PHB1 |
| 56 | CASP3 | 286 | ATL1 | 516 | GAS5 | 746 | TEX2 | 976 | RPTOR | 1206 | DUOXA2 |
| 57 | DERL1 | 287 | TEX264 | 517 | LINC02605 | 747 | SCP2 | 977 | TRAPPC4 | 1207 | NAMPT |
| 58 | MAPK14 | 288 | FOXO1 | 518 | POGLUT2 | 748 | GABARAPL1 | 978 | ILVBL | 1208 | IGF1R |
| 59 | LMAN1 | 289 | UBL4A | 519 | CASP2 | 749 | RELA | 979 | EGR1 | 1209 | PTPA |
| 60 | NFE2L1 | 290 | TRAF2 | 520 | ALG2 | 750 | RPL10 | 980 | XIST | 1210 | THBS4 |
| 61 | PPP1R15A | 291 | DDOST | 521 | SPAST | 751 | PLP1 | 981 | PTPN11 | 1211 | PCK1 |
| 62 | TNF | 292 | GBA1 | 522 | STARD3 | 752 | F9 | 982 | TM7SF2 | 1212 | PMAIP1 |
| 63 | MIR7-3HG | 293 | FKBP5 | 523 | PACS2 | 753 | BTRC | 983 | EIF5A | 1213 | AVPR2 |
| 64 | TXNDC12 | 294 | PINK1 | 524 | APEX1 | 754 | POMT2 | 984 | BMP2 | 1214 | EIF2S2 |
| 65 | PRKN | 295 | NOTCH3 | 525 | ALDH3A2 | 755 | CGRRF1 | 985 | YTHDF1 | 1215 | TRA |
| 66 | G3BP1 | 296 | SAR1B | 526 | KNG1 | 756 | NOD1 | 986 | SERPINI1 | 1216 | GAA |
| 67 | DERL2 | 297 | CREB1 | 527 | IL10 | 757 | HACD2 | 987 | EIF2A | 1217 | ADCYAP1 |
| 68 | CAT | 298 | CAV1 | 528 | HFE | 758 | SFTA3 | 988 | RPLP0 | 1218 | PDLIM1 |
| 69 | BCAP31 | 299 | BNIP1 | 529 | PRDX3 | 759 | NR1H2 | 989 | JAGN1 | 1219 | G6PC2 |
| 70 | CALM1 | 300 | CCDC47 | 530 | TRIM21 | 760 | KDSR | 990 | GHRL | 1220 | NSFL1C |
| 71 | PSEN1 | 301 | SEC61G | 531 | USE1 | 761 | FURIN | 991 | ATXN2L | 1221 | CYP17A1 |
| 72 | DNAJC10 | 302 | TTN | 532 | DSP | 762 | TMCO1 | 992 | HSPG2 | 1222 | SYNCRIP |
| 73 | CASP4 | 303 | EIF4G1 | 533 | RNY5 | 763 | HAX1 | 993 | CYP3A4 | 1223 | IGF2BP2 |
| 74 | MAP3K5 | 304 | MEG3 | 534 | HCRT | 764 | PPP1CB | 994 | CIRBP | 1224 | IAPP |
| 75 | TMX2-CTNND1 | 305 | DDX3X | 535 | GSTM1 | 765 | MAPKAP1 | 995 | TUBA1B | 1225 | CDKAL1 |
| 76 | HYOU1 | 306 | MYOC | 536 | MAP2K4 | 766 | FAM120A | 996 | UBE2D1 | 1226 | LPGAT1 |
| 77 | CERNA3 | 307 | CRYAB | 537 | CAMK2G | 767 | APOA1 | 997 | SRP9 | 1227 | MTHFR |
| 78 | DNAJB9 | 308 | LOC110806262 | 538 | TRP-AGG2-5 | 768 | GRIN2B | 998 | GJB2 | 1228 | NPPB |
| 79 | LMNA | 309 | SGK1 | 539 | MAOA | 769 | OSBPL3 | 999 | HUWE1 | 1229 | EEF1D |
| 80 | BAX | 310 | TNFRSF10B | 540 | EEF1A1 | 770 | SELENOF | 1000 | XIAP | 1230 | UBA1 |
| 81 | ATF6B | 311 | HTRA2 | 541 | CLN8 | 771 | TMEM258 | 1001 | COL4A1 | 1231 | EOGT |
| 82 | INS | 312 | FOXO3 | 542 | EP300 | 772 | PPM1L | 1002 | LCN2 | 1232 | NR1H3 |
| 83 | AMFR | 313 | PRDX4 | 543 | OSBP | 773 | PARP16 | 1003 | CREBBP | 1233 | RPL18 |
| 84 | PDIA4 | 314 | UGGT1 | 544 | VCAM1 | 774 | LRRC59 | 1004 | RIC3 | 1234 | IL18 |
| 85 | ITPR1 | 315 | MAPKAPK2 | 545 | EDN1 | 775 | SGPP1 | 1005 | ABCA1 | 1235 | CRHR2 |
| 86 | LNPK | 316 | MALAT1 | 546 | CDK1 | 776 | SLC39A7 | 1006 | GORASP1 | 1236 | RPS2 |
| 87 | H6PD | 317 | SLN | 547 | PPP1R15B | 777 | PCNA | 1007 | PIK3C3 | 1237 | SLC22A5 |
| 88 | DNAJC3 | 318 | NR3C2 | 548 | CLN6 | 778 | PML | 1008 | SSR3 | 1238 | SFPQ |
| 89 | SIL1 | 319 | SLC35B1 | 549 | CDKN2A | 779 | TERT | 1009 | RPS6 | 1239 | ABCC6 |
| 90 | HSPA4 | 320 | SEC22B | 550 | RYR3 | 780 | PLOD3 | 1010 | RACK1 | 1240 | TAB2 |
| 91 | TXNDC5 | 321 | RNF186 | 551 | ANXA5 | 781 | SOD3 | 1011 | ABCD4 | 1241 | PLA2G4C |
| 92 | BAG6 | 322 | RSAD2 | 552 | DHCR7 | 782 | CDK5RAP3 | 1012 | SRXN1 | 1242 | ZDHHC2 |
| 93 | SEC23A | 323 | SOAT1 | 553 | CUL3 | 783 | REEP1 | 1013 | MLEC | 1243 | ABCG1 |
| 94 | SMAD5-AS1 | 324 | COPB1 | 554 | DMPK | 784 | CALHM1 | 1014 | CYB5R4 | 1244 | ACSL3 |
| 95 | STIM1 | 325 | UBQLN1 | 555 | MZB1 | 785 | MX1 | 1015 | PRKD1 | 1245 | FASLG |
| 96 | WFS1 | 326 | DNM1L | 556 | GABARAPL2 | 786 | F10 | 1016 | HTR2A | 1246 | DAB2IP |
| 97 | EIF2AK2 | 327 | FOXRED2 | 557 | FMR1 | 787 | PLOD1 | 1017 | BSG | 1247 | CYP19A1 |
| 98 | STIP1 | 328 | EGF | 558 | IGF1 | 788 | PWAR4 | 1018 | CLCN1 | 1248 | AGPAT1 |
| 99 | CLU | 329 | EIF2AK4 | 559 | UGGT2 | 789 | BCL2L10 | 1019 | CES1 | 1249 | EMC9 |
| 100 | CFTR | 330 | G6PD | 560 | MICA | 790 | ANK1 | 1020 | CPQ | 1250 | MYH7 |
| 101 | SCN5A | 331 | CDKN1A | 561 | PIK3R1 | 791 | TRAPPC2 | 1021 | LACC1 | 1251 | MIR27A |
| 102 | MTOR | 332 | BRSK2 | 562 | STAU1 | 792 | PPP1R12A | 1022 | VAMP7 | 1252 | ACER3 |
| 103 | MAPK1 | 333 | JPH2 | 563 | CSNK2B | 793 | SOAT2 | 1023 | IL2 | 1253 | PSMC3 |
| 104 | DNAJB11 | 334 | SEC24C | 564 | PPARGC1A | 794 | GRIN2A | 1024 | SDHB | 1254 | RUVBL2 |
| 105 | FOS | 335 | SEC24D | 565 | VHL | 795 | MYDGF | 1025 | CD74 | 1255 | RPL27 |
| 106 | G3BP2 | 336 | PDCD6 | 566 | BRCA1 | 796 | SSR2 | 1026 | ATG2A | 1256 | TSC2 |
| 107 | SIRT1 | 337 | CAV3 | 567 | RINT1 | 797 | HTR1A | 1027 | GRP | 1257 | AKAP6 |
| 108 | MANF | 338 | HIF1A | 568 | FN1 | 798 | MIR21 | 1028 | ITGB1 | 1258 | NR4A1 |
| 109 | MAN1B1 | 339 | P4HTM | 569 | ORMDL3 | 799 | DELE1 | 1029 | PIGBOS1 | 1259 | GTF2I |
| 110 | SLC6A4 | 340 | GBF1 | 570 | ICMT | 800 | HSPA6 | 1030 | SGPP2 | 1260 | HSD11B2 |
| 111 | ANK2 | 341 | FICD | 571 | STT3A | 801 | UBQLN2 | 1031 | PXN | 1261 | CDK2 |
| 112 | CXCL8 | 342 | RNF183 | 572 | BET1 | 802 | NOTCH2 | 1032 | DOLK | 1262 | NFYA |
| 113 | HSPA1A | 343 | ADIPOQ | 573 | SLC2A1 | 803 | UGT1A10 | 1033 | TRPV1 | 1263 | TPO |
| 114 | HSPA8 | 344 | SNORD15A | 574 | TF | 804 | COPE | 1034 | ETFA | 1264 | NCL |
| 115 | ATF3 | 345 | CNIH4 | 575 | PON2 | 805 | SUMF2 | 1035 | TRAPPC11 | 1265 | MIR381 |
| 116 | HSP90AA1 | 346 | TMEM259 | 576 | EMC7 | 806 | NPM1 | 1036 | ENTPD5 | 1266 | CACNA1A |
| 117 | DERL3 | 347 | PRKAA2 | 577 | HSD11B1 | 807 | AGTR1 | 1037 | FBXO6 | 1267 | CEBPA |
| 118 | EMSLR | 348 | NOD2 | 578 | CAPN3 | 808 | CTSD | 1038 | APAF1 | 1268 | PLCG1 |
| 119 | HSF1 | 349 | PCSK9 | 579 | RAC1 | 809 | COMP | 1039 | SORT1 | 1269 | UBE2N |
| 120 | STING1 | 350 | ATL2 | 580 | PCSK1 | 810 | TRAPPC5 | 1040 | BCAP29 | 1270 | PDYN |
| 121 | VAPB | 351 | G6PC1 | 581 | KCNE1 | 811 | ANKS4B | 1041 | CASP1 | 1271 | C3orf52 |
| 122 | OXSR1 | 352 | CACNA1C | 582 | KCNJ11 | 812 | ZFYVE27 | 1042 | SRI | 1272 | GPAA1 |
| 123 | SEL1L | 353 | SLC37A4 | 583 | TMX2 | 813 | SCARA3 | 1043 | RAD51 | 1273 | ARV1 |
| 124 | CREB3 | 354 | TFG | 584 | GRIA1 | 814 | TLR9 | 1044 | SNHG1 | 1274 | CERS6 |
| 125 | CREB3L1 | 355 | CYB5A | 585 | SMPD4 | 815 | CNR1 | 1045 | PPP1R3A | 1275 | MT-RNR2 |
| 126 | PARK7 | 356 | RPN2 | 586 | REEP4 | 816 | VKORC1 | 1046 | FLT3 | 1276 | IGF2BP3 |
| 127 | IL6 | 357 | HSPD1 | 587 | EMC2 | 817 | UNC93B1 | 1047 | OPA1 | 1277 | MYBPC3 |
| 128 | SELENOS | 358 | COMT | 588 | GANAB | 818 | VDAC1 | 1048 | TRAPPC3 | 1278 | PSMB5 |
| 129 | HSPB1 | 359 | PREB | 589 | YIF1B | 819 | DGAT1 | 1049 | TBXAS1 | 1279 | ERBB2 |
| 130 | ERGIC3 | 360 | TMED10 | 590 | JPH4 | 820 | GORASP2 | 1050 | DAPK1 | 1280 | PLAT |
| 131 | CYCS | 361 | GET1 | 591 | PRDX5 | 821 | CYP1A2 | 1051 | CRELD2 | 1281 | EXT1 |
| 132 | RTN4 | 362 | TMX3 | 592 | UBA52 | 822 | TRAPPC12 | 1052 | HNRNPA1 | 1282 | COPG1 |
| 133 | JUN | 363 | SGTA | 593 | NUPR1 | 823 | COL2A1 | 1053 | DPM2 | 1283 | BCR |
| 134 | GSR | 364 | GAPDH | 594 | TRPV4 | 824 | CKB | 1054 | FGFR3 | 1284 | FKBP2 |
| 135 | VAPA | 365 | EDEM2 | 595 | TYR | 825 | PRKACA | 1055 | CDIPT | 1285 | RAB3GAP1 |
| 136 | H19 | 366 | RNF185 | 596 | GRAMD1A | 826 | ACTB | 1056 | PTGES3 | 1286 | MIF |
| 137 | CRH | 367 | STX18 | 597 | TRE-TTC3-1 | 827 | PPARA | 1057 | EIF2B1 | 1287 | VMA21 |
| 138 | EPM2A | 368 | RETREG3 | 598 | FAS | 828 | PIK3CA | 1058 | GRIN1 | 1288 | MIR451A |
| 139 | AKT1 | 369 | MDM2 | 599 | TRV-AAC1-4 | 829 | TRPA1 | 1059 | RPL6 | 1289 | SORL1 |
| 140 | PARP1 | 370 | NOTCH1 | 600 | GPER1 | 830 | BCHE | 1060 | CLGN | 1290 | TMEM30A |
| 141 | TXN | 371 | CYP2E1 | 601 | VMP1 | 831 | ZFAND1 | 1061 | S100A1 | 1291 | KRT8 |
| 142 | UFL1 | 372 | COPA | 602 | GOLGA2 | 832 | SLC30A5 | 1062 | LPIN1 | 1292 | RNF19B |
| 143 | SEC61B | 373 | GPX7 | 603 | INSR | 833 | ALG14 | 1063 | NOTCH4 | 1293 | QDPR |
| 144 | YIPF5 | 374 | CLN3 | 604 | ARL6IP5 | 834 | TFRC | 1064 | TRPM4 | 1294 | DAD1 |
| 145 | KCNH2 | 375 | BCL2L11 | 605 | GRAMD1B | 835 | BRCA2 | 1065 | TRIP11 | 1295 | TRAPPC9 |
| 146 | PDIA2 | 376 | REEP5 | 606 | SHC1 | 836 | NDUFS4 | 1066 | RTN2 | 1296 | PIGA |
| 147 | CASQ1 | 377 | CBS | 607 | EPO | 837 | RPA1 | 1067 | MAPK8IP1 | 1297 | BGLAP |
| 148 | SOD2 | 378 | ALG1 | 608 | KCNJ5 | 838 | WLS | 1068 | ACTG1 | 1298 | PNKD |
| 149 | CKAP4 | 379 | TAP2 | 609 | OXTR | 839 | E2F1 | 1069 | RBX1 | 1299 | SCYL2 |
| 150 | DNAH8 | 380 | HSPA1B | 610 | EIF4E | 840 | G6PC3 | 1070 | LRPAP1 | 1300 | CREB3L4 |
| 151 | EDEM1 | 381 | BECN1 | 611 | ZW10 | 841 | PMM2 | 1071 | PRKRA | 1301 | CYP2B6 |
| 152 | SREBF1 | 382 | FKRP | 612 | NPLOC4 | 842 | MMP9 | 1072 | ELOVL2 | 1302 | GOLPH3 |
| 153 | BDNF | 383 | OSBPL8 | 613 | P3H4 | 843 | ZMPSTE24 | 1073 | DNAJC16 | 1303 | LPL |
| 154 | SIGMAR1 | 384 | RCN2 | 614 | STRIT1 | 844 | SRP72 | 1074 | GCLC | 1304 | MUC1 |
| 155 | SEC61A1 | 385 | SAR1A | 615 | RNF5 | 845 | NGF | 1075 | AQP2 | 1305 | CLPTM1L |
| 156 | PRNP | 386 | CASP7 | 616 | UBXN8 | 846 | MIR193BHG | 1076 | MGAT2 | 1306 | MIR210 |
| 157 | TMEM33 | 387 | TECRL | 617 | LEP | 847 | ABCC8 | 1077 | MSRB1 | 1307 | SELENOT |
| 158 | ERLIN2 | 388 | EDEM3 | 618 | F2 | 848 | TNFRSF1A | 1078 | TTF2 | 1308 | NCSTN |
| 159 | MIA3 | 389 | SRC | 619 | PCSK6 | 849 | HSD17B12 | 1079 | MIAT | 1309 | SPCS1 |
| 160 | SEC31A | 390 | USO1 | 620 | ATXN2 | 850 | FITM2 | 1080 | B2M | 1310 | PLP2 |
| 161 | RPN1 | 391 | SHH | 621 | PITPNB | 851 | ATXN3 | 1081 | GOLGB1 | 1311 | EPAS1 |
| 162 | ABL1 | 392 | PRKCD | 622 | CYB5R3 | 852 | JPH3 | 1082 | CDNF | 1312 | ARRB1 |
| 163 | ATP13A1 | 393 | MOSPD2 | 623 | MAP2K3 | 853 | AKAP9 | 1083 | LRP6 | 1313 | USP13 |
| 164 | NR3C1 | 394 | CRP | 624 | PABPC1 | 854 | SPPL2C | 1084 | ANXA2 | 1314 | ADAM17 |
| 165 | TGFB1 | 395 | DHCR24 | 625 | NCK1 | 855 | DUSP19 | 1085 | CEMIP | 1315 | DIPK1C |
| 166 | KEAP1 | 396 | SRPRA | 626 | UFD1 | 856 | GBA2 | 1086 | RHO | 1316 | CRAT |
| 167 | TMED4 | 397 | BBC3 | 627 | CERT1 | 857 | SPCS2 | 1087 | ZDHHC4 | 1317 | ALOX5 |
| 168 | SURF4 | 398 | LDLR | 628 | PTPN2 | 858 | STT3B | 1088 | DSG2 | 1318 | SPPL3 |
| 169 | GET3 | 399 | CSNK2A1 | 629 | PRL | 859 | DYSF | 1089 | PPP1R10 | 1319 | TRPC1 |
| 170 | CASP9 | 400 | MAP2K6 | 630 | MIR34C | 860 | TNFSF10 | 1090 | POFUT1 | 1320 | FLOT1 |
| 171 | SELENON | 401 | TG | 631 | DPM1 | 861 | CAPN2 | 1091 | ABCC1 | 1321 | UBE4B |
| 172 | DDRGK1 | 402 | CYBA | 632 | TRPM2 | 862 | MCFD2 | 1092 | BIRC2 | 1322 | MARCHF6 |
| 173 | STX17 | 403 | RMRP | 633 | LPCAT3 | 863 | TLR2 | 1093 | YY1 | 1323 | ABCA7 |
| 174 | UBC | 404 | CALM3 | 634 | HDAC6 | 864 | UBE2D3 | 1094 | ADAMTS13 | 1324 | MAN1A2 |
| 175 | BSCL2 | 405 | QRICH1 | 635 | CCND1 | 865 | SEC31B | 1095 | ANP32A | 1325 | SERINC3 |
| 176 | SERPINA1 | 406 | GSTP1 | 636 | DSPP | 866 | PWAR1 | 1096 | ACP1 | 1326 | COPS5 |
| 177 | SEC13 | 407 | SDF2L1 | 637 | OMA1 | 867 | RAF1 | 1097 | COLGALT1 | 1327 | FAF1 |
| 178 | SREBF2 | 408 | AQP11 | 638 | NDRG1 | 868 | GRINA | 1098 | ELOVL1 | 1328 | FBXO17 |
| 179 | RETREG1 | 409 | ZDHHC6 | 639 | CP | 869 | CALU | 1099 | GLUD1 | 1329 | NIBAN1 |
| 180 | MIA2 | 410 | GPX1 | 640 | PLEKHF2 | 870 | SIRT3 | 1100 | ADAMTSL1 | 1330 | CALR3 |
| 181 | PTPN1 | 411 | PRDX2 | 641 | XK | 871 | CYP1B1 | 1101 | ATG9A | 1331 | FBXO27 |
| 182 | MBTPS1 | 412 | TMED7 | 642 | HLA-C | 872 | MPPE1 | 1102 | ELOVL7 | 1332 | UBXN10 |
| 183 | MPO | 413 | RAB6A | 643 | KRAS | 873 | KCNMA1 | 1103 | EZH2 | 1333 | CTH |
| 184 | LMAN2 | 414 | SCD | 644 | RETREG2 | 874 | VAMP2 | 1104 | VCPIP1 | 1334 | TTC23L |
| 185 | TMX1 | 415 | TMEM117 | 645 | TUG1 | 875 | ICAM1 | 1105 | TUBB | 1335 | BHLHA15 |
| 186 | PDIA6 | 416 | PRDX1 | 646 | LBR | 876 | LNPEP | 1106 | UBXN1 | 1336 | ELAVL4 |
| 187 | PGR-AS1 | 417 | MOGS | 647 | BOK | 877 | YWHAZ | 1107 | SCYL1 | 1337 | FCGR2B |
| 188 | CASP8 | 418 | CYBB | 648 | PIGN | 878 | EEF2 | 1108 | ALG13 | 1338 | ALOX15 |
| 189 | INSIG1 | 419 | PTGIS | 649 | PRKCA | 879 | SNTA1 | 1109 | RPL26 | 1339 | FBXO2 |
| 190 | STX5 | 420 | HLA-A | 650 | MCL1 | 880 | EPM2A-DT | 1110 | GH1 | 1340 | FGF21 |
| 191 | HSPA9 | 421 | TMED9 | 651 | SMPD1 | 881 | LOC129997381 | 1111 | NCK2 | 1341 | AFF4 |
| 192 | CEBPB | 422 | TIA1 | 652 | PKM | 882 | TECR | 1112 | PRKCSH | 1342 | RNF175 |
| 193 | TRA-TGC7-1 | 423 | PKD2 | 653 | ITPR2 | 883 | ERMARD | 1113 | TBL2 | 1343 | USP25 |
| 194 | SQSTM1 | 424 | MICB | 654 | RAB18 | 884 | CHEK1 | 1114 | EMC8 | 1344 | NRBF2 |
| 195 | MAPK10 | 425 | NPC1 | 655 | NOS1 | 885 | CDC42 | 1115 | LMBRD1 | 1345 | NCCRP1 |
| 196 | TMBIM6 | 426 | HTT | 656 | SLC39A14 | 886 | GOSR2 | 1116 | MIR199A1 | 1346 | PDX1 |
| 197 | PTGS2 | 427 | PDZD8 | 657 | KPNB1 | 887 | BID | 1117 | IKBKG | 1347 | KCNJ8 |
| 198 | CREB3L2 | 428 | PPARG | 658 | ADRB2 | 888 | GOSR1 | 1118 | BAG1 | 1348 | MIR200C |
| 199 | MBTPS2 | 429 | HSPA13 | 659 | DST | 889 | AKR1B1 | 1119 | SDHA | 1349 | MARCKS |
| 200 | TMED2 | 430 | UGT1A1 | 660 | JAK2 | 890 | MAN1A1 | 1120 | CYP21A2 | 1350 | MAGEA3 |
| 201 | MAPT | 431 | ESR1 | 661 | PTGS1 | 891 | RAB4B-EGLN2 | 1121 | IGFBP1 | 1351 | FAM8A1 |
| 202 | BCL2L1 | 432 | YIF1A | 662 | BRAF | 892 | ATP1A1 | 1122 | HSPA2 | 1352 | PIK3R2 |
| 203 | VWF | 433 | UBE2G2 | 663 | PIGK | 893 | TMEM43 | 1123 | TANGO2 | 1353 | ANKZF1 |
| 204 | APOB | 434 | TMEM208 | 664 | IGF2BP1 | 894 | ARSA | 1124 | LGALS3 | 1354 | PPP2CB |
| 205 | SCAP | 435 | CYP1A1 | 665 | SFTPC | 895 | DAXX | 1125 | RHBDF1 | 1355 | RNF121 |
| 206 | IL1B | 436 | CREBRF | 666 | RCN1 | 896 | CFLAR | 1126 | PLA2G4A | 1356 | PARP6 |
| 207 | VEGFA | 437 | MAP1LC3B | 667 | PROC | 897 | SSR4 | 1127 | TMCC2 | 1357 | PSMC6 |
| 208 | LRRK2 | 438 | TMCC1 | 668 | DRD2 | 898 | UFC1 | 1128 | HSPB8 | 1358 | MAN1C1 |
| 209 | UBE2J1 | 439 | TMEM214 | 669 | UCP2 | 899 | VTRNA1-1 | 1129 | UGT1A | 1359 | RCN3 |
| 210 | KCNQ1 | 440 | LMAN2L | 670 | EMC3 | 900 | CHKB-CPT1B | 1130 | LOC106627981 | 1360 | RHBDD2 |
| 211 | HMGCR | 441 | IER3IP1 | 671 | RAB2A | 901 | ADAM10 | 1131 | PIGT | 1361 | CREBZF |
| 212 | NLRP3 | 442 | EIF2B5 | 672 | DPAGT1 | 902 | ATP7A | 1132 | RPL4 | 1362 | RASGRF1 |
| 213 | ALB | 443 | PPP1CA | 673 | SRPRB | 903 | NOL3 | 1133 | PSMD4 | 1363 | RASGRF2 |
| 214 | SELENOK | 444 | EMC10 | 674 | POR | 904 | COL1A1 | 1134 | TM4SF20 | 1364 | TRIM25 |
| 215 | PSEN2 | 445 | HRC | 675 | C9orf72 | 905 | DNAJB1 | 1135 | ELN | 1365 | RNF103 |
| 216 | RTN3 | 446 | MYC | 676 | PKD1 | 906 | ZFAND2B | 1136 | NLRP1 | 1366 | TMUB2 |
| 217 | SNCA | 447 | TRC-GCA24-1 | 677 | ATF2 | 907 | IL24 | 1137 | H2AX | 1367 | CHAC1 |
| 218 | EIF2AK1 | 448 | CLCC1 | 678 | MGST1 | 908 | HP | 1138 | TFEB | 1368 | PARP8 |
| 219 | CREB3L3 | 449 | AUP1 | 679 | UBXN4 | 909 | RET | 1139 | AGT | 1369 | SEL1L2 |
| 220 | PLN | 450 | SRP68 | 680 | MIR34A | 910 | NSF | 1140 | DHX9 | 1370 | UBXN6 |
| 221 | DLEU2 | 451 | MFN2 | 681 | UBB | 911 | USP10 | 1141 | TLX1NB | 1371 | TMUB1 |
| 222 | TAPBP | 452 | MAP2K7 | 682 | RPL11 | 912 | STARD3NL | 1142 | EPHX1 | 1372 | ATG10 |
| 223 | ERLIN1 | 453 | FAM20C | 683 | MIR133B | 913 | SGF29 | 1143 | AGO1 | 1373 | SRPX |
| 224 | NOS3 | 454 | PIK3CG | 684 | TMED1 | 914 | SPTLC1 | 1144 | RARA | 1374 | TMTC4 |
| 225 | HLA-B | 455 | RAB10 | 685 | EMC4 | 915 | TRAF6 | 1145 | MET | 1375 | RNFT2 |
| 226 | HMGB1 | 456 | TOR1B | 686 | NBAS | 916 | DNAJA1 | 1146 | PTK2 | 1376 | STC2 |
| 227 | SEC24A | 457 | AIFM1 | 687 | AGR3 | 917 | PLOD2 | 1147 | LRP1 | 1377 | USP14 |
| 228 | NHLRC1 | 458 | PIEZO1 | 688 | PSMD2 | 918 | UGT1A7 | 1148 | SLX1A-SULT1A3 | 1378 | TMEM129 |
| 229 | TARDBP | 459 | NPY | 689 | YBX1 | 919 | LONP1 | 1149 | PIGH | 1379 | UBE4A |
| 230 | SESN2 | 460 | INPP5K | 690 | SRP14 | 920 | SVIP | 1150 | PDHA1 | 1380 | FBXO44 |

**Table S2** | TIS genes

| 1 | CCL1 | 141 | CTNS | 281 | TMEPAI | 421 | CXCL13 |
| --- | --- | --- | --- | --- | --- | --- | --- |
| 2 | EBI3 | 142 | F13A1 | 282 | ADARB1 | 422 | HEY1 |
| 3 | INDO | 143 | FABP4 | 283 | AF107846 | 423 | HIST1H4K |
| 4 | LAMP3 | 144 | FZD2 | 284 | ALDH1B1 | 424 | ICA1 |
| 5 | OAS3 | 145 | GSTT1 | 285 | APBB2 | 425 | KCNK5 |
| 6 | ABCB4 | 146 | GUCA1A | 286 | ATL2 | 426 | KIAA1324 |
| 7 | BACH2 | 147 | HS3ST2 | 287 | BCL2 | 427 | MAF |
| 8 | BCL11A | 148 | LMAN2L | 288 | CDC5L | 428 | MAGEH1 |
| 9 | BLK | 149 | MMP12 | 289 | FGF18 | 429 | MKL2 |
| 10 | BLNK | 150 | MS4A6A | 290 | FUT5 | 430 | MYO6 |
| 11 | CCR9 | 151 | NUDT9 | 291 | FZR1 | 431 | MYO7A |
| 12 | CD19 | 152 | PDXK | 292 | GAGE2A | 432 | PASK |
| 13 | CD72 | 153 | PPARG | 293 | IGFBP5 | 433 | PDCD1 |
| 14 | COCH | 154 | PREP | 294 | KANK2 | 434 | POMT1 |
| 15 | CR2 | 155 | RAP1GAP | 295 | LDB3 | 435 | PTPN13 |
| 16 | DTNB | 156 | SLC26A6 | 296 | MAPRE3 | 436 | PVALB |
| 17 | FCRL2 | 157 | SLC7A8 | 297 | MCM3AP | 437 | SH3TC1 |
| 18 | GLDC | 158 | SYT17 | 298 | MRC2 | 438 | SIRPG |
| 19 | GNG7 | 159 | TACSTD2 | 299 | NCR1 | 439 | SLC7A10 |
| 20 | HLA-DOB | 160 | TM7SF4 | 300 | PDLIM4 | 440 | SMAD1 |
| 21 | HLA-DQA1 | 161 | VASH1 | 301 | PRX | 441 | ST8SIA1 |
| 22 | IGHA1 | 162 | APOE | 302 | PSMD4 | 442 | STK39 |
| 23 | IGHG1 | 163 | ATG7 | 303 | RP5-886K2.1 | 443 | THADA |
| 24 | IGHM | 164 | BCAT1 | 304 | SGMS1 | 444 | TOX |
| 25 | IGKC | 165 | CCL7 | 305 | SLC30A5 | 445 | TSHR |
| 26 | IGL | 166 | CD163 | 306 | SMEK1 | 446 | ZNF764 |
| 27 | KIAA0125 | 167 | CD68 | 307 | SPN | 447 | C1orf61 |
| 28 | MEF2C | 168 | CD84 | 308 | TBXA2R | 448 | CD160 |
| 29 | MICAL3 | 169 | CHI3L1 | 309 | TCTN2 | 449 | FEZ1 |
| 30 | MS4A1 | 170 | CHIT1 | 310 | TINAGL1 | 450 | TARP |
| 31 | OSBPL10 | 171 | CLEC5A | 311 | TRPV6 | 451 | TRD |
| 32 | PNOC | 172 | COL8A2 | 312 | XCL1 | 452 | TRGV9 |
| 33 | QRSL1 | 173 | COLEC12 | 313 | XCL2 | 453 | APBB2 |
| 34 | SCN3A | 174 | CTSK | 314 | ZNF205 | 454 | APOD |
| 35 | SLC15A2 | 175 | CXCL5 | 315 | ZNF528 | 455 | ATP9A |
| 36 | SPIB | 176 | CYBB | 316 | ZNF747 | 456 | BST2 |
| 37 | TCL1A | 177 | DNASE2B | 317 | IL3RA | 457 | BTG3 |
| 38 | TNFRSF17 | 178 | EMP1 | 318 | BCL11B | 458 | CCL4 |
| 39 | ABT1 | 179 | FDX1 | 319 | CD2 | 459 | CD38 |
| 40 | AES | 180 | FN1 | 320 | CD28 | 460 | CD70 |
| 41 | APBA2 | 181 | GM2A | 321 | CD3D | 461 | CMAH |
| 42 | ARHGAP8 | 182 | GPC4 | 322 | CD3E | 462 | CSF2 |
| 43 | C12orf47 | 183 | KAL1 | 323 | CD3G | 463 | CTLA4 |
| 44 | C19orf6 | 184 | MARCO | 324 | CD6 | 464 | DGKI |
| 45 | C4orf15 | 185 | ME1 | 325 | CD96 | 465 | DOK5 |
| 46 | CAMLG | 186 | MS4A4A | 326 | GIMAP5 | 466 | DPP4 |
| 47 | CD8A | 187 | MSR1 | 327 | ITM2A | 467 | DUSP5 |
| 48 | CD8B | 188 | PCOLCE2 | 328 | LCK | 468 | EGFL6 |
| 49 | CDKN2AIP | 189 | PTGDS | 329 | NCALD | 469 | GGT1 |
| 50 | DNAJB1 | 190 | RAI14 | 330 | PRKCQ | 470 | HBEGF |
| 51 | FLT3LG | 191 | SCARB2 | 331 | SH2D1A | 471 | IFNG |
| 52 | GADD45A | 192 | SCG5 | 332 | SKAP1 | 472 | IL12RB2 |
| 53 | GZMM | 193 | SGMS1 | 333 | TRA | 473 | IL22 |
| 54 | KLF9 | 194 | SULT1C2 | 334 | TRAC | 474 | LRP8 |
| 55 | LEPROTL1 | 195 | ABCC4 | 335 | TRAT1 | 475 | LRRN3 |
| 56 | LIME1 | 196 | ADCYAP1 | 336 | TRBC1 | 476 | LTA |
| 57 | MYST3 | 197 | CALB2 | 337 | ANP32B | 477 | SGCB |
| 58 | PF4 | 198 | CEACAM8 | 338 | ASF1A | 478 | SYNGR3 |
| 59 | PPP1R2 | 199 | CMA1 | 339 | ATF2 | 479 | ZBTB32 |
| 60 | PRF1 | 200 | CPA3 | 340 | BATF | 480 | IL17A |
| 61 | PRR5 | 201 | CTSG | 341 | C13orf34 | 481 | IL17RA |
| 62 | RBM3 | 202 | ELA2 | 342 | CD28 | 482 | RORC |
| 63 | SF1 | 203 | GATA2 | 343 | DDX50 | 483 | ADCY1 |
| 64 | SFRS7 | 204 | HDC | 344 | FAM111A | 484 | AHI1 |
| 65 | SLC16A7 | 205 | HPGD | 345 | FRYL | 485 | ANK1 |
| 66 | TBCC | 206 | HPGDS | 346 | GOLGA8A | 486 | BIRC5 |
| 67 | THUMPD1 | 207 | KIT | 347 | ICOS | 487 | CDC25C |
| 68 | TMC6 | 208 | LINC01140 | 348 | ITM2A | 488 | CDC7 |
| 69 | TSC22D3 | 209 | MAOB | 349 | LRBA | 489 | CENPF |
| 70 | VAMP2 | 210 | MLPH | 350 | NAP1L4 | 490 | CXCR6 |
| 71 | ZEB1 | 211 | MPO | 351 | NUP107 | 491 | DHFR |
| 72 | ZFP36L2 | 212 | MS4A2 | 352 | PHF10 | 492 | EVI5 |
| 73 | ZNF22 | 213 | NR0B1 | 353 | PPP2R5C | 493 | GATA3 |
| 74 | ZNF609 | 214 | PPM1H | 354 | RPA1 | 494 | GSTA4 |
| 75 | ZNF91 | 215 | PRG2 | 355 | SEC24C | 495 | HELLS |
| 76 | APBA2 | 216 | PTGS1 | 356 | SLC25A12 | 496 | IL26 |
| 77 | APOL3 | 217 | SCG2 | 357 | SRSF10 | 497 | LAIR2 |
| 78 | CTSW | 218 | SIGLEC6 | 358 | TRA | 498 | LIMA1 |
| 79 | DUSP2 | 219 | SLC18A2 | 359 | UBE2L3 | 499 | MB |
| 80 | GNLY | 220 | SLC24A3 | 360 | YME1L1 | 500 | MICAL2 |
| 81 | GZMA | 221 | TAL1 | 361 | AQP3 | 501 | NEIL3 |
| 82 | GZMH | 222 | TPSAB1 | 362 | ATF7IP | 502 | PHEX |
| 83 | KLRB1 | 223 | TPSB2 | 363 | ATM | 503 | PMCH |
| 84 | KLRD1 | 224 | VWA5A | 364 | CASP8 | 504 | PTGIS |
| 85 | KLRF1 | 225 | ALPL | 365 | CDC14A | 505 | SLC39A14 |
| 86 | KLRK1 | 226 | BST1 | 366 | CEP68 | 506 | SMAD2 |
| 87 | NKG7 | 227 | CD93 | 367 | CLUAP1 | 507 | SNRPD1 |
| 88 | RORA | 228 | CEACAM3 | 368 | CREBZF | 508 | WDHD1 |
| 89 | RUNX3 | 229 | CREB5 | 369 | CYLD | 509 | FOXP3 |
| 90 | SIGIRR | 230 | CRISPLD2 | 370 | DOCK9 | 510 | CDH5 |
| 91 | WHAMMP3 | 231 | CSF3R | 371 | FAM153B | 511 | ELTD1 |
| 92 | ZBTB16 | 232 | CYP4F3 | 372 | FOXP1 | 512 | CLEC14A |
| 93 | CCL13 | 233 | DYSF | 373 | FYB | 513 | LDB2 |
| 94 | CCL17 | 234 | FCAR | 374 | HNRPH1 | 514 | ECSCR |
| 95 | CCL22 | 235 | FCGR3B | 375 | INPP4B | 515 | MYCT1 |
| 96 | CD209 | 236 | FLJ11151 | 376 | KLF12 | 516 | RHOJ |
| 97 | HSD11B1 | 237 | FPR1 | 377 | LOC441155 | 517 | VWF |
| 98 | NPR1 | 238 | FPRL1 | 378 | MAP3K1 | 518 | TIE1 |
| 99 | PPFIBP2 | 239 | G0S2 | 379 | MLL | 519 | KDR |
| 100 | ABHD2 | 240 | HIST1H2BC | 380 | N4BP2L2-IT2 | 520 | ESAM |
| 101 | ACACB | 241 | HPSE | 381 | NEFL | 521 | PTPRB |
| 102 | C9orf156 | 242 | IL8RA | 382 | NFATC3 | 522 | GPR116 |
| 103 | CAT | 243 | IL8RB | 383 | PCM1 | 523 | SPARCL1 |
| 104 | CCR3 | 244 | KCNJ15 | 384 | PCNX | 524 | EMCN |
| 105 | CLC | 245 | LILRB2 | 385 | PDXDC2 | 525 | ROBO4 |
| 106 | CYSLTR2 | 246 | MGAM | 386 | PHC3 | 526 | ENG |
| 107 | EMR1 | 247 | MME | 387 | POLR2J2 | 527 | TEK |
| 108 | EPN2 | 248 | PDE4B | 388 | PSPC1 | 528 | S1PR1 |
| 109 | GALC | 249 | S100A12 | 389 | REPS1 | 529 | A2M |
| 110 | GPR44 | 250 | SIGLEC5 | 390 | RPP38 | 530 | JAM2 |
| 111 | HES1 | 251 | SLC22A4 | 391 | SLC7A6 | 531 | COL15A1 |
| 112 | HIST1H1C | 252 | SLC25A37 | 392 | SNRPN | 532 | PECAM1 |
| 113 | HRH4 | 253 | TECPR2 | 393 | ST3GAL1 | 533 | CALCRL |
| 114 | IGSF2 | 254 | TNFRSF10C | 394 | STX16 | 534 | CLEC3B |
| 115 | IL5RA | 255 | VNN3 | 395 | TIMM8A | 535 | PLVAP |
| 116 | KBTBD11 | 256 | DUSP4 | 396 | TRAF3IP3 | 536 | RGS5 |
| 117 | KCNH2 | 257 | FOXJ1 | 397 | TXK | 537 | LRRC32 |
| 118 | LRP5L | 258 | LPCAT4 | 398 | TXLNGY | 538 | EBF1 |
| 119 | MYO15B | 259 | MADD | 399 | USP9Y | 539 | ADCY4 |
| 120 | RCOR3 | 260 | 6-Mar | 400 | AKT3 | 540 | ACVRL1 |
| 121 | RNASE2 | 261 | MPPED1 | 401 | C7orf54 | 541 | GPR124 |
| 122 | RRP12 | 262 | MUC3B | 402 | CCR2 | 542 | APLNR |
| 123 | SIAH1 | 263 | NIBP | 403 | DDX17 | 543 | TM4SF18 |
| 124 | SMPD3 | 264 | PLA2G6 | 404 | EWSR1 | 544 | GNG11 |
| 125 | SYNJ1 | 265 | RRAD | 405 | FLI1 | 545 | CNRIP1 |
| 126 | TGIF1 | 266 | XCL1 | 406 | GDPD5 | 546 | ZNF423 |
| 127 | THBS1 | 267 | EDG8 | 407 | LTK | 547 | GIMAP8 |
| 128 | THBS4 | 268 | FLJ20699 | 408 | MEFV | 548 | PDGFD |
| 129 | TIPARP | 269 | GTF3C1 | 409 | NFATC4 | 549 | ITGA9 |
| 130 | TKTL1 | 270 | GZMB | 410 | PRKY | 550 | EDNRB |
| 131 | ABCG2 | 271 | IL21R | 411 | TBC1D5 | 551 | HLA-A |
| 132 | BLVRB | 272 | KIR2DL3 | 412 | TBCD | 552 | HLA-B |
| 133 | CARD9 | 273 | KIR2DS1 | 413 | TRA | 553 | HLA-C |
| 134 | CD1A | 274 | KIR2DS2 | 414 | VIL2 | 554 | B2M |
| 135 | CD1B | 275 | KIR2DS5 | 415 | B3GAT1 | 555 | TAP1 |
| 136 | CD1C | 276 | KIR3DL1 | 416 | BLR1 | 556 | TAP2 |
| 137 | CD1E | 277 | KIR3DL2 | 417 | C18orf1 | 557 | TAPBP |
| 138 | CH25H | 278 | KIR3DL3 | 418 | CDK5R1 |  |  |
| 139 | CLEC10A | 279 | KIR3DS1 | 419 | CHGB |  |  |
| 140 | CSF1R | 280 | SPON2 | 420 | CHI3L2 |  |  |

**Table S3** | APM genes

| 1 | PSMB5 | 7 | TAP1 | 13 | PDIA3 |
| --- | --- | --- | --- | --- | --- |
| 2 | PSMB6 | 8 | TAP2 | 14 | TAPBP |
| 3 | PSMB7 | 9 | ERAP1 | 15 | B2M |
| 4 | PSMB8 | 10 | ERAP2 | 16 | HLA-A |
| 5 | PSMB9 | 11 | CANX | 17 | HLA-B |
| 6 | PSMB10 | 12 | CALR | 18 | HLA-C |

**Table S4** | CYT genes

| 1 | GZMA |
| --- | --- |
| 2 | PRF1 |

**Table S5** | GET genes

| 1 | CD27 | 8 | CD200R1 | 15 | BATF |
| --- | --- | --- | --- | --- | --- |
| 2 | SIRPG | 9 | CD80 | 16 | TIGIT |
| 3 | CXCR6 | 10 | TNS3 | 17 | VDR |
| 4 | ICOS | 11 | KIR2DL4 | 18 | CTLA4 |
| 5 | RUNX2 | 12 | ZBED2 | 19 | LAG3 |
| 6 | TNFRSF9 | 13 | TNIP3 | 20 | KLRB1 |
| 7 | CD70 | 14 | SEMA4A | 21 | TNFRSF18 |

**Table S6** | Probes for unsupervised clustering.

| 1 | cg24760581 | 186 | cg21542078 | 371 | cg27205904 | 556 | cg18633684 | 741 | cg24015249 |
| --- | --- | --- | --- | --- | --- | --- | --- | --- | --- |
| 2 | cg09102257 | 187 | cg06638433 | 372 | cg00269606 | 557 | cg06734406 | 742 | cg16664570 |
| 3 | cg20776829 | 188 | cg13798970 | 373 | cg07177756 | 558 | cg16101346 | 743 | cg20000562 |
| 4 | cg24506221 | 189 | cg22861561 | 374 | cg07227926 | 559 | cg14018363 | 744 | cg25722465 |
| 5 | cg07017374 | 190 | cg21844749 | 375 | cg12101586 | 560 | cg17844121 | 745 | cg23378033 |
| 6 | cg01558040 | 191 | cg26364899 | 376 | cg08892386 | 561 | cg11773720 | 746 | cg01520924 |
| 7 | cg20448717 | 192 | cg26447697 | 377 | cg03789645 | 562 | cg21052814 | 747 | cg18998365 |
| 8 | cg14399369 | 193 | cg04105966 | 378 | cg27366984 | 563 | cg25682171 | 748 | cg01244043 |
| 9 | cg23491124 | 194 | cg00098799 | 379 | cg02726263 | 564 | cg19598416 | 749 | cg11539424 |
| 10 | cg00043095 | 195 | cg04899175 | 380 | cg26537639 | 565 | cg15852258 | 750 | cg05156901 |
| 11 | cg14075454 | 196 | cg26286839 | 381 | cg03436208 | 566 | cg11037477 | 751 | cg13143529 |
| 12 | cg04470054 | 197 | cg09980058 | 382 | cg01875838 | 567 | cg03762483 | 752 | cg08186005 |
| 13 | cg25884711 | 198 | cg09025501 | 383 | cg10963543 | 568 | cg02453828 | 753 | cg15820961 |
| 14 | cg06539717 | 199 | cg06902379 | 384 | cg12944530 | 569 | cg18650716 | 754 | cg11251858 |
| 15 | cg11574174 | 200 | cg21858255 | 385 | cg23256802 | 570 | cg14506366 | 755 | cg14660839 |
| 16 | cg04194494 | 201 | cg05026102 | 386 | cg07376033 | 571 | cg17096289 | 756 | cg27619291 |
| 17 | cg02099572 | 202 | cg10253465 | 387 | cg05224770 | 572 | cg17174980 | 757 | cg04789225 |
| 18 | cg02899346 | 203 | cg19031575 | 388 | cg19155735 | 573 | cg12840719 | 758 | cg00803088 |
| 19 | cg18373158 | 204 | cg23480619 | 389 | cg21120661 | 574 | cg10770023 | 759 | cg21024264 |
| 20 | cg03787864 | 205 | cg00054525 | 390 | cg27095527 | 575 | cg05929864 | 760 | cg12882103 |
| 21 | cg16964348 | 206 | cg12742178 | 391 | cg11681321 | 576 | cg13634501 | 761 | cg10791023 |
| 22 | cg00675569 | 207 | cg05966641 | 392 | cg25299895 | 577 | cg18515591 | 762 | cg10792923 |
| 23 | cg20249919 | 208 | cg13415207 | 393 | cg08106706 | 578 | cg04207385 | 763 | cg19949241 |
| 24 | cg04548815 | 209 | cg14765933 | 394 | cg15759721 | 579 | cg23625084 | 764 | cg24105634 |
| 25 | cg04705620 | 210 | cg12207120 | 395 | cg23685650 | 580 | cg08166587 | 765 | cg07738730 |
| 26 | cg08208480 | 211 | cg19537719 | 396 | cg12668482 | 581 | cg27348223 | 766 | cg16867657 |
| 27 | cg18912855 | 212 | cg21946299 | 397 | cg10002850 | 582 | cg06839377 | 767 | cg09975620 |
| 28 | cg05585821 | 213 | cg21097881 | 398 | cg13506670 | 583 | cg19369424 | 768 | cg03405789 |
| 29 | cg02647388 | 214 | cg16503611 | 399 | cg14311811 | 584 | cg06782676 | 769 | cg19728345 |
| 30 | cg09895920 | 215 | cg26001287 | 400 | cg23693487 | 585 | cg21163347 | 770 | cg03762694 |
| 31 | cg13910785 | 216 | cg04106006 | 401 | cg06756211 | 586 | cg21475834 | 771 | cg10208370 |
| 32 | cg11694519 | 217 | cg03443590 | 402 | cg01681236 | 587 | cg09994117 | 772 | cg08845336 |
| 33 | cg20858454 | 218 | cg09533293 | 403 | cg23400446 | 588 | cg03423957 | 773 | cg25217583 |
| 34 | cg06829830 | 219 | cg26614816 | 404 | cg19249107 | 589 | cg08370787 | 774 | cg03761750 |
| 35 | cg04879832 | 220 | cg24436906 | 405 | cg05215925 | 590 | cg04633225 | 775 | cg12496211 |
| 36 | cg01141940 | 221 | cg19418273 | 406 | cg02144298 | 591 | cg00772000 | 776 | cg19825483 |
| 37 | cg13759674 | 222 | cg25677261 | 407 | cg21515023 | 592 | cg24394631 | 777 | cg01465364 |
| 38 | cg14142965 | 223 | cg16306978 | 408 | cg14276286 | 593 | cg05440824 | 778 | cg19517653 |
| 39 | cg02192967 | 224 | cg24718971 | 409 | cg17342132 | 594 | cg11625005 | 779 | cg13316625 |
| 40 | cg23905789 | 225 | cg27615388 | 410 | cg21773872 | 595 | cg23125970 | 780 | cg18336854 |
| 41 | cg12883479 | 226 | cg25820257 | 411 | cg14022022 | 596 | cg08260245 | 781 | cg19594666 |
| 42 | cg14335894 | 227 | cg21408061 | 412 | cg24642523 | 597 | cg03531100 | 782 | cg16212074 |
| 43 | cg03310087 | 228 | cg25412831 | 413 | cg11981868 | 598 | cg20697984 | 783 | cg01952742 |
| 44 | cg21981270 | 229 | cg27023597 | 414 | cg05655647 | 599 | cg06570931 | 784 | cg24188561 |
| 45 | cg14200170 | 230 | cg09511741 | 415 | cg04222582 | 600 | cg00691240 | 785 | cg07822788 |
| 46 | cg17494781 | 231 | cg17573292 | 416 | cg06173395 | 601 | cg08855903 | 786 | cg00171421 |
| 47 | cg25276694 | 232 | cg15174834 | 417 | cg09797202 | 602 | cg25637655 | 787 | cg15172739 |
| 48 | cg17480035 | 233 | cg24724428 | 418 | cg10673740 | 603 | cg14203613 | 788 | cg13373703 |
| 49 | cg26093711 | 234 | cg22879515 | 419 | cg25941151 | 604 | cg18104285 | 789 | cg26973714 |
| 50 | cg27085741 | 235 | cg17283601 | 420 | cg01640150 | 605 | cg18721249 | 790 | cg15926585 |
| 51 | cg15384598 | 236 | cg22847691 | 421 | cg00920970 | 606 | cg11850773 | 791 | cg08642292 |
| 52 | cg02328010 | 237 | cg18106668 | 422 | cg14132731 | 607 | cg24735307 | 792 | cg01052512 |
| 53 | cg10598353 | 238 | cg00930615 | 423 | cg09419486 | 608 | cg15194943 | 793 | cg05796704 |
| 54 | cg11990980 | 239 | cg11946459 | 424 | cg07718303 | 609 | cg24931138 | 794 | cg16204066 |
| 55 | cg16831085 | 240 | cg19622755 | 425 | cg24885417 | 610 | cg11256152 | 795 | cg00158333 |
| 56 | cg24377694 | 241 | cg23353432 | 426 | cg06111374 | 611 | cg15501942 | 796 | cg16243756 |
| 57 | cg10384245 | 242 | cg12308054 | 427 | cg02985568 | 612 | cg05937873 | 797 | cg24888257 |
| 58 | cg23029363 | 243 | cg15722404 | 428 | cg24610236 | 613 | cg12266049 | 798 | cg15456502 |
| 59 | cg21035222 | 244 | cg01583131 | 429 | cg00963071 | 614 | cg16179182 | 799 | cg08538032 |
| 60 | cg04616797 | 245 | cg19156875 | 430 | cg07324702 | 615 | cg12986110 | 800 | cg09195389 |
| 61 | cg00888521 | 246 | cg03422911 | 431 | cg14486477 | 616 | cg08091439 | 801 | cg15375772 |
| 62 | cg16448058 | 247 | cg14411266 | 432 | cg04458919 | 617 | cg06618866 | 802 | cg05914150 |
| 63 | cg14904662 | 248 | cg27243389 | 433 | cg06565641 | 618 | cg17119907 | 803 | cg22502206 |
| 64 | cg02365862 | 249 | cg13210403 | 434 | cg20463862 | 619 | cg05393736 | 804 | cg14741143 |
| 65 | cg09556042 | 250 | cg25147026 | 435 | cg00840332 | 620 | cg19779211 | 805 | cg00565882 |
| 66 | cg05505450 | 251 | cg00193021 | 436 | cg13931925 | 621 | cg19384106 | 806 | cg00051662 |
| 67 | cg11917734 | 252 | cg15070677 | 437 | cg09580336 | 622 | cg13911501 | 807 | cg16602097 |
| 68 | cg14502484 | 253 | cg05366024 | 438 | cg08194989 | 623 | cg06493386 | 808 | cg07118376 |
| 69 | cg00119181 | 254 | cg27312312 | 439 | cg19407459 | 624 | cg19611392 | 809 | cg10782668 |
| 70 | cg22293140 | 255 | cg07181702 | 440 | cg01966878 | 625 | cg22346380 | 810 | cg09367967 |
| 71 | cg14362312 | 256 | cg21351483 | 441 | cg05997059 | 626 | cg12758687 | 811 | cg23725454 |
| 72 | cg07336840 | 257 | cg04525892 | 442 | cg21341586 | 627 | cg16514085 | 812 | cg19788250 |
| 73 | cg12865888 | 258 | cg24245418 | 443 | cg02623400 | 628 | cg21122656 | 813 | cg04675204 |
| 74 | cg14186641 | 259 | cg05166490 | 444 | cg11512365 | 629 | cg21608605 | 814 | cg10513437 |
| 75 | cg24394819 | 260 | cg12631351 | 445 | cg12227210 | 630 | cg19696441 | 815 | cg18749617 |
| 76 | cg16836311 | 261 | cg20145598 | 446 | cg24530264 | 631 | cg26888153 | 816 | cg07338205 |
| 77 | cg24600221 | 262 | cg04276626 | 447 | cg01418388 | 632 | cg15108590 | 817 | cg27096416 |
| 78 | cg26635219 | 263 | cg15309264 | 448 | cg15811668 | 633 | cg12901182 | 818 | cg08129449 |
| 79 | cg20811857 | 264 | cg06515144 | 449 | cg13643509 | 634 | cg15279308 | 819 | cg06471491 |
| 80 | cg08708229 | 265 | cg02344868 | 450 | cg17352276 | 635 | cg05129479 | 820 | cg09416203 |
| 81 | cg08843517 | 266 | cg03078488 | 451 | cg08086724 | 636 | cg02413285 | 821 | cg13713537 |
| 82 | cg12314713 | 267 | cg21331088 | 452 | cg23715728 | 637 | cg19112186 | 822 | cg19099850 |
| 83 | cg04846243 | 268 | cg18132851 | 453 | cg15452573 | 638 | cg05785947 | 823 | cg02331910 |
| 84 | cg19098763 | 269 | cg05383619 | 454 | cg26669806 | 639 | cg18938907 | 824 | cg16243197 |
| 85 | cg17535691 | 270 | cg06945523 | 455 | cg01005968 | 640 | cg26534425 | 825 | cg26720913 |
| 86 | cg19709083 | 271 | cg11680055 | 456 | cg17616554 | 641 | cg19096849 | 826 | cg20578783 |
| 87 | cg02478379 | 272 | cg21849393 | 457 | cg17629148 | 642 | cg18729298 | 827 | cg15373592 |
| 88 | cg05488523 | 273 | cg18258770 | 458 | cg03338754 | 643 | cg25592413 | 828 | cg14544583 |
| 89 | cg11445109 | 274 | cg17167852 | 459 | cg01770296 | 644 | cg10635145 | 829 | cg02027123 |
| 90 | cg02658690 | 275 | cg17076592 | 460 | cg12522722 | 645 | cg22932804 | 830 | cg23951474 |
| 91 | cg25514328 | 276 | cg12466613 | 461 | cg03886192 | 646 | cg05057777 | 831 | cg19730814 |
| 92 | cg23514324 | 277 | cg05744675 | 462 | cg11661868 | 647 | cg12477716 | 832 | cg26132320 |
| 93 | cg15676015 | 278 | cg25285646 | 463 | cg20548888 | 648 | cg09858022 | 833 | cg16879574 |
| 94 | cg03764092 | 279 | cg20818407 | 464 | cg09697084 | 649 | cg11853320 | 834 | cg25924694 |
| 95 | cg03650429 | 280 | cg19485202 | 465 | cg09405790 | 650 | cg14346243 | 835 | cg13775050 |
| 96 | cg13678973 | 281 | cg00963169 | 466 | cg25067162 | 651 | cg17286640 | 836 | cg06225476 |
| 97 | cg17067544 | 282 | cg06880615 | 467 | cg14119706 | 652 | cg07860673 | 837 | cg12669271 |
| 98 | cg06404175 | 283 | cg11251827 | 468 | cg21234561 | 653 | cg11274962 | 838 | cg15690475 |
| 99 | cg24242823 | 284 | cg10862468 | 469 | cg20627916 | 654 | cg16669455 | 839 | cg15517609 |
| 100 | cg17059658 | 285 | cg19677203 | 470 | cg01070854 | 655 | cg03987506 | 840 | cg05846851 |
| 101 | cg16319578 | 286 | cg18125573 | 471 | cg14703605 | 656 | cg09949775 | 841 | cg12150039 |
| 102 | cg22549041 | 287 | cg21800193 | 472 | cg22155039 | 657 | cg02452966 | 842 | cg17429236 |
| 103 | cg03125427 | 288 | cg11052780 | 473 | cg01035160 | 658 | cg26234223 | 843 | cg06430387 |
| 104 | cg27107893 | 289 | cg18640030 | 474 | cg22370006 | 659 | cg26666107 | 844 | cg09197492 |
| 105 | cg14091954 | 290 | cg15085086 | 475 | cg01357135 | 660 | cg15646817 | 845 | cg17445802 |
| 106 | cg26807107 | 291 | cg18375860 | 476 | cg14250048 | 661 | cg22584138 | 846 | cg15100426 |
| 107 | cg12935170 | 292 | cg05765580 | 477 | cg06299307 | 662 | cg05850327 | 847 | cg21633105 |
| 108 | cg26764761 | 293 | cg02704535 | 478 | cg09307564 | 663 | cg01561259 | 848 | cg06264984 |
| 109 | cg09139047 | 294 | cg26937434 | 479 | cg07914084 | 664 | cg09992387 | 849 | cg12030690 |
| 110 | cg05158615 | 295 | cg08125539 | 480 | cg15887283 | 665 | cg06861044 | 850 | cg19807685 |
| 111 | cg02515217 | 296 | cg03296248 | 481 | cg17299636 | 666 | cg06700060 | 851 | cg04906740 |
| 112 | cg08573687 | 297 | cg05789595 | 482 | cg09149648 | 667 | cg10986462 | 852 | cg20716703 |
| 113 | cg23401796 | 298 | cg01786715 | 483 | cg04176995 | 668 | cg06357305 | 853 | cg20668321 |
| 114 | cg10406526 | 299 | cg13012916 | 484 | cg26036029 | 669 | cg18226166 | 854 | cg11661187 |
| 115 | cg25598086 | 300 | cg03563308 | 485 | cg19968840 | 670 | cg07181406 | 855 | cg08065229 |
| 116 | cg18397450 | 301 | cg22304399 | 486 | cg02293410 | 671 | cg27321931 | 856 | cg01718447 |
| 117 | cg09678212 | 302 | cg16193278 | 487 | cg23109897 | 672 | cg13330671 | 857 | cg25508319 |
| 118 | cg14722290 | 303 | cg07690455 | 488 | cg14669921 | 673 | cg11404544 | 858 | cg08215831 |
| 119 | cg15397593 | 304 | cg05852760 | 489 | cg21117668 | 674 | cg05487134 | 859 | cg15519474 |
| 120 | cg14081884 | 305 | cg03988778 | 490 | cg26460471 | 675 | cg26876834 | 860 | cg03127174 |
| 121 | cg27135692 | 306 | cg13940693 | 491 | cg09582351 | 676 | cg15126273 | 861 | cg00689010 |
| 122 | cg21191514 | 307 | cg14827929 | 492 | cg01971483 | 677 | cg14262937 | 862 | cg22354782 |
| 123 | cg08729686 | 308 | cg24891846 | 493 | cg07010337 | 678 | cg08332908 | 863 | cg14245102 |
| 124 | cg00601836 | 309 | cg08741898 | 494 | cg19037167 | 679 | cg04837280 | 864 | cg00532477 |
| 125 | cg16439198 | 310 | cg10740902 | 495 | cg04025739 | 680 | cg11025609 | 865 | cg03182958 |
| 126 | cg03307465 | 311 | cg18211014 | 496 | cg26002259 | 681 | cg13583454 | 866 | cg22327175 |
| 127 | cg12145080 | 312 | cg00321709 | 497 | cg03505654 | 682 | cg14738823 | 867 | cg17794358 |
| 128 | cg13216112 | 313 | cg04026937 | 498 | cg10784511 | 683 | cg04232935 | 868 | cg10057033 |
| 129 | cg13469471 | 314 | cg05194426 | 499 | cg20917241 | 684 | cg23929344 | 869 | cg11796996 |
| 130 | cg08404221 | 315 | cg16449699 | 500 | cg11398323 | 685 | cg02415992 | 870 | cg06483739 |
| 131 | cg01407254 | 316 | cg26755097 | 501 | cg00401233 | 686 | cg23174662 | 871 | cg24960291 |
| 132 | cg09626894 | 317 | cg19366147 | 502 | cg15057434 | 687 | cg00735923 | 872 | cg24046411 |
| 133 | cg03817911 | 318 | cg03935060 | 503 | cg06849723 | 688 | cg10829004 | 873 | cg21212505 |
| 134 | cg09405169 | 319 | cg14300347 | 504 | cg24375221 | 689 | cg12046677 | 874 | cg07584093 |
| 135 | cg18878992 | 320 | cg08177833 | 505 | cg10511332 | 690 | cg13863078 | 875 | cg16373862 |
| 136 | cg02072495 | 321 | cg19474267 | 506 | cg21410080 | 691 | cg19528338 | 876 | cg02286533 |
| 137 | cg26299169 | 322 | cg13315147 | 507 | cg08189615 | 692 | cg15465743 | 877 | cg02589828 |
| 138 | cg11969813 | 323 | cg06613263 | 508 | cg14395444 | 693 | cg20154618 | 878 | cg14705695 |
| 139 | cg08757742 | 324 | cg26298737 | 509 | cg16369400 | 694 | cg14489474 | 879 | cg07619683 |
| 140 | cg09072162 | 325 | cg09806262 | 510 | cg18998670 | 695 | cg09925075 | 880 | cg25909396 |
| 141 | cg06497198 | 326 | cg20818806 | 511 | cg24216893 | 696 | cg06959053 | 881 | cg04561261 |
| 142 | cg18984983 | 327 | cg23221090 | 512 | cg16590635 | 697 | cg08931917 | 882 | cg06875305 |
| 143 | cg11859607 | 328 | cg11783901 | 513 | cg10572274 | 698 | cg23736297 | 883 | cg01077846 |
| 144 | cg17331296 | 329 | cg07589972 | 514 | cg16472060 | 699 | cg14535332 | 884 | cg27501748 |
| 145 | cg08269402 | 330 | cg12695586 | 515 | cg14487577 | 700 | cg25022311 | 885 | cg09208540 |
| 146 | cg10908116 | 331 | cg10698654 | 516 | cg04056576 | 701 | cg18064631 | 886 | cg01459453 |
| 147 | cg13064658 | 332 | cg13928417 | 517 | cg02426611 | 702 | cg08575537 | 887 | cg27131953 |
| 148 | cg19108881 | 333 | cg27370558 | 518 | cg27608999 | 703 | cg27041424 | 888 | cg02529544 |
| 149 | cg11651220 | 334 | cg06573644 | 519 | cg15919612 | 704 | cg13923094 | 889 | cg13528344 |
| 150 | cg17204129 | 335 | cg19451698 | 520 | cg06799305 | 705 | cg10502244 | 890 | cg21398111 |
| 151 | cg01947066 | 336 | cg06649410 | 521 | cg07376282 | 706 | cg04885072 | 891 | cg04212229 |
| 152 | cg22892328 | 337 | cg05372242 | 522 | cg26656751 | 707 | cg12578166 | 892 | cg20749005 |
| 153 | cg16457786 | 338 | cg17395064 | 523 | cg23881278 | 708 | cg14027161 | 893 | cg00350503 |
| 154 | cg16113692 | 339 | cg22022067 | 524 | cg26135849 | 709 | cg10731794 | 894 | cg16331823 |
| 155 | cg19480198 | 340 | cg01321962 | 525 | cg00155593 | 710 | cg09901733 | 895 | cg17953636 |
| 156 | cg16083711 | 341 | cg17992056 | 526 | cg23869439 | 711 | cg15929698 | 896 | cg23533270 |
| 157 | cg01881939 | 342 | cg02086457 | 527 | cg12781915 | 712 | cg13782781 | 897 | cg25509184 |
| 158 | cg19517525 | 343 | cg26278103 | 528 | cg03885646 | 713 | cg01286133 | 898 | cg01804281 |
| 159 | cg20615879 | 344 | cg13185030 | 529 | cg24710631 | 714 | cg27294268 | 899 | cg06539449 |
| 160 | cg23991636 | 345 | cg26335127 | 530 | cg17066594 | 715 | cg14929208 | 900 | cg09101894 |
| 161 | cg12578486 | 346 | cg19521832 | 531 | cg11056055 | 716 | cg10125465 | 901 | cg05671385 |
| 162 | cg17520909 | 347 | cg00584422 | 532 | cg02892357 | 717 | cg09477407 | 902 | cg23973115 |
| 163 | cg20923245 | 348 | cg06738356 | 533 | cg06922606 | 718 | cg13495204 | 903 | cg14112075 |
| 164 | cg18792022 | 349 | cg09378783 | 534 | cg21109025 | 719 | cg23624321 | 904 | cg14993491 |
| 165 | cg09508356 | 350 | cg26477549 | 535 | cg18996663 | 720 | cg20372821 | 905 | cg23602832 |
| 166 | cg01157404 | 351 | cg14032089 | 536 | cg03716852 | 721 | cg07381788 | 906 | cg14328115 |
| 167 | cg01331992 | 352 | cg12407526 | 537 | cg23914842 | 722 | cg13621396 | 907 | cg19416417 |
| 168 | cg14594187 | 353 | cg07733851 | 538 | cg17263974 | 723 | cg26374305 | 908 | cg26796518 |
| 169 | cg09365002 | 354 | cg18433146 | 539 | cg23409074 | 724 | cg00421848 | 909 | cg25565730 |
| 170 | cg27498387 | 355 | cg11583287 | 540 | cg14972143 | 725 | cg12833465 | 910 | cg19311375 |
| 171 | cg14849140 | 356 | cg19439706 | 541 | cg12614105 | 726 | cg13209481 | 911 | cg19426944 |
| 172 | cg10069493 | 357 | cg01698567 | 542 | cg09414638 | 727 | cg03598159 | 912 | cg26839117 |
| 173 | cg26606256 | 358 | cg06684850 | 543 | cg09332604 | 728 | cg02281970 | 913 | cg08309183 |
| 174 | cg22366350 | 359 | cg24770985 | 544 | cg15442907 | 729 | cg27370471 | 914 | cg03085312 |
| 175 | cg19837601 | 360 | cg18609044 | 545 | cg23037321 | 730 | cg24685601 | 915 | cg09580214 |
| 176 | cg15633390 | 361 | cg13518792 | 546 | cg04825215 | 731 | cg07172242 | 916 | cg21993547 |
| 177 | cg13767940 | 362 | cg23623667 | 547 | cg09884423 | 732 | cg23000806 | 917 | cg04382470 |
| 178 | cg14711366 | 363 | cg00588621 | 548 | cg22719623 | 733 | cg18183624 | 918 | cg09126559 |
| 179 | cg14621254 | 364 | cg13570656 | 549 | cg17529716 | 734 | cg09149894 | 919 | cg06632027 |
| 180 | cg25009451 | 365 | cg08303146 | 550 | cg16749456 | 735 | cg17025741 | 920 | cg01940810 |
| 181 | cg19136632 | 366 | cg00661777 | 551 | cg12838303 | 736 | cg13582457 | 921 | cg17405178 |
| 182 | cg13413286 | 367 | cg18466674 | 552 | cg02643433 | 737 | cg06838584 | 922 | cg01612095 |
| 183 | cg11008866 | 368 | cg11064395 | 553 | cg11363972 | 738 | cg24131595 | 923 | cg15421962 |
| 184 | cg05598562 | 369 | cg19962990 | 554 | cg11854928 | 739 | cg21467935 | 924 | cg19790294 |
| 185 | cg20891501 | 370 | cg25444386 | 555 | cg14984684 | 740 | cg20118643 | 925 | cg08823027 |

**Table S7** | Performance metrics for consensus matrix

| Metric | Value | | | | |
| --- | --- | --- | --- | --- | --- |
|  | s.d. > 0.15  (n = 3732) | s.d. > 0.18  (n= 1745) | s.d. > 0.20  (n = 925) | s.d. > 0.22  (n = 419) | s.d. > 0.25  (n = 121) |
| cophenetic | 0.971 | 0.986 | 0.992 | 0.992 | 0.987 |
| silhouette.consensus | 0.887 | 0.927 | 0.953 | 0.954 | 0.929 |
| dispersion | 0.873 | 0.898 | 0.928 | 0.938 | 0.912 |
| evar | 0.931 | 0.909 | 0.883 | 0.864 | 0.849 |

**Table S8** | The feature subset including 33 probes.

| 1 | cg07017374 | 12 | cg08757742 | 23 | cg22022067 |
| --- | --- | --- | --- | --- | --- |
| 2 | cg20448717 | 13 | cg13798970 | 24 | cg19439706 |
| 3 | cg23491124 | 14 | cg09980058 | 25 | cg25444386 |
| 4 | cg02899346 | 15 | cg06902379 | 26 | cg07718303 |
| 5 | cg02647388 | 16 | cg12631351 | 27 | cg01035160 |
| 6 | cg14200170 | 17 | cg25285646 | 28 | cg17529716 |
| 7 | cg02365862 | 18 | cg03563308 | 29 | cg13634501 |
| 8 | cg03650429 | 19 | cg19366147 | 30 | cg24735307 |
| 9 | cg14091954 | 20 | cg09806262 | 31 | cg23000806 |
| 10 | cg25598086 | 21 | cg12695586 | 32 | cg07738730 |
| 11 | cg13469471 | 22 | cg17395064 | 33 | cg16212074 |

**Table S9** | Overall classification performance in 5-fold cross-validation on the training set, with 95% confidence intervals.

| Metric | Value | 95% CI |
| --- | --- | --- |
| Accuracy | 0.913 | 0.858-0.969 |
| Precision | 0.929 | 0.879-0.979 |
| Recall | 0.920 | 0.867-0.973 |
| F1-score | 0.921 | 0.867-0.976 |

**Table S10** | Overall classification performance on the independent test set, with bootstrap 95% confidence intervals.

| Metric | Value | 95% CI |
| --- | --- | --- |
| Accuracy | 0.924 | 0.867-0.971 |
| Precision | 0.928 | 0.865-0.978 |
| Recall | 0.925 | 0.868-0.973 |
| F1-score | 0.925 | 0.864-0.975 |

**Table S11** | Contingency tables and cell-wise residual statistics for comparisons between ERS-related methylation subtypes and established GBM classification systems. Expected counts were calculated under the null hypothesis of independence. Positive adjusted standardized residuals indicate over-representation, whereas negative values indicate under-representation. As a rule of thumb, absolute adjusted standardized residual values greater than 1.96 suggest non-random enrichment or depletion.

A. ERS-related methylation subtypes vs Ceccarelli classification

| **ERS subtype** | **Reference class** | **Observed** | **Expected** | **Pearson residual** | **Adjusted standardized residual** |
| --- | --- | --- | --- | --- | --- |
| Subtype 1 | LGm1 | 0 | 1.68 | -1.3 | -1.54 |
| Subtype 1 | LGm2 | 0 | 0.28 | -0.53 | -0.62 |
| Subtype 1 | LGm4 | 85 | 45.84 | 5.78 | 9.38 |
| Subtype 1 | LGm5 | 12 | 32.99 | -3.65 | -5.3 |
| Subtype 1 | LGm6 | 0 | 16.21 | -4.03 | -5.2 |
| Subtype 2 | LGm1 | 0 | 2.47 | -1.57 | -2.07 |
| Subtype 2 | LGm2 | 0 | 0.41 | -0.64 | -0.84 |
| Subtype 2 | LGm4 | 65 | 67.59 | -0.31 | -0.56 |
| Subtype 2 | LGm5 | 67 | 48.63 | 2.63 | 4.23 |
| Subtype 2 | LGm6 | 11 | 23.9 | -2.64 | -3.77 |
| Subtype 3 | LGm1 | 4 | 1.14 | 2.68 | 3 |
| Subtype 3 | LGm2 | 0 | 0.19 | -0.44 | -0.49 |
| Subtype 3 | LGm4 | 12 | 31.19 | -3.44 | -5.26 |
| Subtype 3 | LGm5 | 28 | 22.44 | 1.17 | 1.6 |
| Subtype 3 | LGm6 | 22 | 11.03 | 3.3 | 4.02 |
| Subtype 4 | LGm1 | 2 | 0.71 | 1.53 | 1.65 |
| Subtype 4 | LGm2 | 1 | 0.12 | 2.57 | 2.74 |
| Subtype 4 | LGm4 | 2 | 19.38 | -3.95 | -5.79 |
| Subtype 4 | LGm5 | 11 | 13.94 | -0.79 | -1.03 |
| Subtype 4 | LGm6 | 25 | 6.85 | 6.93 | 8.09 |

B. ERS-related methylation subtypes vs Capper classification

| **ERS subtype** | **Reference class** | **Observed** | **Expected** | **Pearson residual** | **Adjusted standardized residual** |
| --- | --- | --- | --- | --- | --- |
| Subtype 1 | GBM_G34 | 0 | 11.46 | -3.39 | -4.25 |
| Subtype 1 | GBM_MES | 1 | 15.65 | -3.7 | -4.76 |
| Subtype 1 | GBM_MID | 0 | 3.91 | -1.98 | -2.38 |
| Subtype 1 | GBM_MYCN | 0 | 4.47 | -2.11 | -2.55 |
| Subtype 1 | GBM_RTK_1 | 26 | 17.89 | 1.92 | 2.5 |
| Subtype 1 | GBM_RTK_2 | 70 | 39.97 | 4.75 | 7.3 |
| Subtype 1 | GBM_RTK_3 | 0 | 3.63 | -1.91 | -2.29 |
| Subtype 2 | GBM_G34 | 0 | 16.9 | -4.11 | -5.71 |
| Subtype 2 | GBM_MES | 55 | 23.08 | 6.65 | 9.46 |
| Subtype 2 | GBM_MID | 0 | 5.77 | -2.4 | -3.2 |
| Subtype 2 | GBM_MYCN | 11 | 6.59 | 1.72 | 2.29 |
| Subtype 2 | GBM_RTK_1 | 3 | 26.37 | -4.55 | -6.57 |
| Subtype 2 | GBM_RTK_2 | 73 | 58.93 | 1.83 | 3.12 |
| Subtype 2 | GBM_RTK_3 | 1 | 5.36 | -1.88 | -2.5 |
| Subtype 3 | GBM_G34 | 0 | 7.8 | -2.79 | -3.3 |
| Subtype 3 | GBM_MES | 0 | 10.65 | -3.26 | -3.96 |
| Subtype 3 | GBM_MID | 14 | 2.66 | 6.95 | 7.88 |
| Subtype 3 | GBM_MYCN | 5 | 3.04 | 1.12 | 1.28 |
| Subtype 3 | GBM_RTK_1 | 35 | 12.17 | 6.54 | 8.05 |
| Subtype 3 | GBM_RTK_2 | 0 | 27.2 | -5.22 | -7.56 |
| Subtype 3 | GBM_RTK_3 | 12 | 2.47 | 6.06 | 6.86 |
| Subtype 4 | GBM_G34 | 41 | 4.84 | 16.43 | 18.63 |
| Subtype 4 | GBM_MES | 0 | 6.62 | -2.57 | -2.99 |
| Subtype 4 | GBM_MID | 0 | 1.65 | -1.29 | -1.4 |
| Subtype 4 | GBM_MYCN | 0 | 1.89 | -1.37 | -1.5 |
| Subtype 4 | GBM_RTK_1 | 0 | 7.56 | -2.75 | -3.24 |
| Subtype 4 | GBM_RTK_2 | 0 | 16.9 | -4.11 | -5.71 |
| Subtype 4 | GBM_RTK_3 | 0 | 1.54 | -1.24 | -1.35 |

**Table S12** | Overall association statistics for comparisons between ERS-related methylation subtypes and established GBM classification systems. Pearson’s chi-square test was used to assess the overall association between ERS-related methylation subtypes and the reference classification systems. Cramér’s V was calculated to estimate association strength. Df indicates the degrees of freedom for the corresponding chi-square test.

| **Comparison system** | **N** | **Table dimension** | **Chi-square** | **df** | **P value** | **Cramér’s V** |
| --- | --- | --- | --- | --- | --- | --- |
| ERS subtypes vs Capper classification | 347 | 4×7 | 641.51 | 18 | 1.42E-124 | 0.785 |
| ERS subtypes vs Ceccarelli classification | 347 | 4×5 | 186.49 | 12 | 1.98E-33 | 0.423 |

**Table S13** | Genomic annotation of 33 CpG probes. The corresponding probe ID, mapped gene, genomic region, and CpG island relationship are shown.

| ID | Gene Name | Genomic Region | CpG_Islands |
| --- | --- | --- | --- |
| cg25285646 | MICA | Body |  |
| cg19366147 | CLN8 | 5'UTR |  |
| cg06902379 | SLN | TSS1500 |  |
| cg13634501 | ANXA2 | 5'UTR | Island |
| cg13798970 | ANXA2 | TSS200 | Island |
| cg09980058 | COMP | Body | Island |
| cg23000806 | PRKDC | Body |  |
| cg16212074 | CREB3L3 | Body | N_Shelf |
| cg12631351 | RASGRF2 | 1st Exon | Island |
| cg25598086 | HSPA2 | TSS1500 | N_Shore |
| cg08757742 | RASGRF2 | TSS200 | Island |
| cg02647388 | DSP | TSS200 | Island |
| cg02365862 | DSP | TSS1500 | Island |
| cg09806262 | ALOX5 | Body | S_Shore |
| cg03563308 | GRP | TSS200 | Island |
| cg07718303 | SPPL3 | Body |  |
| cg17529716 | MICA | Body |  |
| cg25444386 | KDR | Body | N_Shore |
| cg14091954 | FLT3 | TSS200 | Island |
| cg01035160 | SNCA | TSS1500;5'UTR;TSS200 | Island |
| cg13469471 | DSP | TSS200 | Island |
| cg07017374 | FLT3 | Body | Island |
| cg19439706 | TRPA1 | TSS200 | Island |
| cg17395064 | TXNRD1 | TSS200 | Island |
| cg20448717 | DSP | 1st Exon;5'UTR | Island |
| cg22022067 | NOTCH4 | Body |  |
| cg02899346 | RTN2 | Body | N_Shore |
| cg07738730 | IGF2BP1 | Body | S_Shore |
| cg03650429 | FBXO17 | Body | Island |
| cg24735307 | TLX1NB | 5'UTR | N_Shore |
| cg23491124 | PMAIP1 | TSS200 | Island |
| cg12695586 | OXTR | 5'UTR | Island |
| cg14200170 | ADCYAP1 | 5'UTR;TSS200 | Island |

**Table S14** | Classification outcomes of TCGA patients.

|  | Sample name | subtype |
| --- | --- | --- |
| 1 | TCGA-76-6192-01A-11D-1697-05 | 2 |
| 2 | TCGA-28-2510-01A-01D-1697-05 | 2 |
| 3 | TCGA-76-4931-01A-01D-1481-05 | 2 |
| 4 | TCGA-76-6656-01A-11D-1844-05 | 2 |
| 5 | TCGA-06-0190-01A-01D-A45W-05 | 2 |
| 6 | TCGA-06-6388-01A-12D-1844-05 | 2 |
| 7 | TCGA-4W-AA9S-01A-11D-A392-05 | 2 |
| 8 | TCGA-19-5958-01A-11D-1697-05 | 2 |
| 9 | TCGA-06-5410-01A-01D-1697-05 | 2 |
| 10 | TCGA-28-5220-01A-01D-1481-05 | 1 |
| 11 | TCGA-74-6573-01A-12D-1844-05 | 2 |
| 12 | TCGA-06-6699-01A-11D-1844-05 | 1 |
| 13 | TCGA-19-5960-01A-11D-1697-05 | 1 |
| 14 | TCGA-76-6285-01A-11D-1697-05 | 3 |
| 15 | TCGA-81-5910-01A-11D-1697-05 | 2 |
| 16 | TCGA-76-6193-01A-11D-1697-05 | 2 |
| 17 | TCGA-06-6697-01A-11D-1844-05 | 2 |
| 18 | TCGA-28-2501-01A-01D-1697-05 | 2 |
| 19 | TCGA-06-6698-01A-11D-1844-05 | 2 |
| 20 | TCGA-76-4929-01A-01D-1481-05 | 3 |
| 21 | TCGA-76-6282-01A-11D-1697-05 | 2 |
| 22 | TCGA-28-5218-01A-01D-1481-05 | 2 |
| 23 | TCGA-32-1980-01A-01D-1697-05 | 2 |
| 24 | TCGA-06-5858-01A-01D-1697-05 | 2 |
| 25 | TCGA-76-4934-01A-01D-1481-05 | 3 |
| 26 | TCGA-06-6693-01A-11D-1844-05 | 1 |
| 27 | TCGA-06-6389-01A-11D-1697-05 | 2 |
| 28 | TCGA-28-5207-01A-01D-1481-05 | 2 |
| 29 | TCGA-19-5950-01A-11D-1697-05 | 2 |
| 30 | TCGA-12-5299-01A-02D-1481-05 | 2 |
| 31 | TCGA-06-5418-01A-01D-1481-05 | 2 |
| 32 | TCGA-74-6575-01A-11D-1844-05 | 2 |
| 33 | TCGA-26-5135-01A-01D-1481-05 | 3 |
| 34 | TCGA-28-5213-01A-01D-1481-05 | 2 |
| 35 | TCGA-06-5415-01A-01D-1481-05 | 1 |
| 36 | TCGA-06-5414-01A-01D-1481-05 | 1 |
| 37 | TCGA-06-5417-01A-01D-1481-05 | 3 |
| 38 | TCGA-19-5956-01A-11D-1697-05 | 3 |
| 39 | TCGA-06-A5U1-01A-11D-A33U-05 | 2 |
| 40 | TCGA-76-4935-01A-01D-1481-05 | 3 |
| 41 | TCGA-06-5413-01A-01D-1697-05 | 2 |
| 42 | TCGA-76-6664-01A-11D-1844-05 | 2 |
| 43 | TCGA-76-6663-01A-11D-1844-05 | 2 |
| 44 | TCGA-74-6577-01A-11D-1844-05 | 2 |
| 45 | TCGA-76-4927-01A-01D-1481-05 | 1 |
| 46 | TCGA-19-4065-01A-01D-2004-05 | 2 |
| 47 | TCGA-06-1806-01A-02D-1844-05 | 2 |
| 48 | TCGA-41-6646-01A-11D-1844-05 | 2 |
| 49 | TCGA-19-5955-01A-11D-1697-05 | 2 |
| 50 | TCGA-06-5408-01A-01D-1697-05 | 2 |
| 51 | TCGA-28-5216-01A-01D-1481-05 | 3 |
| 52 | TCGA-14-1402-01A-01D-A45W-05 | 1 |
| 53 | TCGA-14-0736-01A-01D-A45W-05 | 2 |
| 54 | TCGA-28-5214-01A-01D-1481-05 | 2 |
| 55 | TCGA-76-4932-01A-01D-1481-05 | 1 |
| 56 | TCGA-26-5139-01A-01D-1481-05 | 2 |
| 57 | TCGA-32-5222-01A-01D-1481-05 | 1 |
| 58 | TCGA-4W-AA9R-01A-11D-A392-05 | 1 |
| 59 | TCGA-06-0650-01A-02D-1697-05 | 2 |
| 60 | TCGA-76-6660-01A-11D-1844-05 | 1 |
| 61 | TCGA-RR-A6KC-01A-31D-A33U-05 | 2 |
| 62 | TCGA-06-A5U0-01A-11D-A33U-05 | 4 |
| 63 | TCGA-28-5219-01A-01D-1481-05 | 3 |
| 64 | TCGA-76-6280-01A-21D-1844-05 | 1 |
| 65 | TCGA-06-6694-01A-12D-1844-05 | 2 |
| 66 | TCGA-06-A6S0-01A-11D-A33U-05 | 1 |
| 67 | TCGA-28-6450-01A-11D-1697-05 | 2 |
| 68 | TCGA-76-6191-01A-12D-1697-05 | 1 |
| 69 | TCGA-76-6662-01A-11D-1844-05 | 2 |
| 70 | TCGA-26-5133-01A-01D-1481-05 | 4 |
| 71 | TCGA-19-A6J4-01A-11D-A33U-05 | 2 |
| 72 | TCGA-41-5651-01A-01D-1697-05 | 3 |
| 73 | TCGA-12-5295-01A-01D-1481-05 | 1 |
| 74 | TCGA-28-5215-01A-01D-1481-05 | 3 |
| 75 | TCGA-06-5412-01A-01D-1697-05 | 2 |
| 76 | TCGA-19-5952-01A-11D-1697-05 | 2 |
| 77 | TCGA-87-5896-01A-01D-1697-05 | 1 |
| 78 | TCGA-06-0211-01A-01D-A45W-05 | 2 |
| 79 | TCGA-06-6391-01A-11D-1697-05 | 2 |
| 80 | TCGA-74-6584-01A-11D-1844-05 | 3 |
| 81 | TCGA-06-5859-01A-01D-1697-05 | 2 |
| 82 | TCGA-06-5416-01A-01D-1481-05 | 3 |
| 83 | TCGA-06-5411-01A-01D-1697-05 | 3 |
| 84 | TCGA-28-5208-01A-01D-1481-05 | 1 |
| 85 | TCGA-74-6578-01A-11D-1844-05 | 2 |
| 86 | TCGA-28-5204-01A-01D-1481-05 | 2 |
| 87 | TCGA-19-1389-01A-01D-A45W-05 | 2 |
| 88 | TCGA-06-0210-01A-01D-A45W-05 | 2 |
| 89 | TCGA-76-6657-01A-11D-1844-05 | 2 |
| 90 | TCGA-19-5951-01A-11D-1697-05 | 2 |
| 91 | TCGA-19-A60I-01A-12D-A33U-05 | 1 |
| 92 | TCGA-06-A6S1-01A-11D-A33U-05 | 2 |
| 93 | TCGA-76-6283-01A-11D-1844-05 | 1 |
| 94 | TCGA-06-5856-01A-01D-1697-05 | 1 |
| 95 | TCGA-06-0152-01A-02D-A45W-05 | 2 |
| 96 | TCGA-74-6581-01A-11D-1844-05 | 1 |
| 97 | TCGA-06-6390-01A-11D-1697-05 | 2 |
| 98 | TCGA-19-5959-01A-11D-1697-05 | 1 |
| 99 | TCGA-06-A7TK-01A-21D-A392-05 | 3 |
| 100 | TCGA-06-0125-01A-01D-A45W-05 | 1 |
| 101 | TCGA-06-6700-01A-12D-1844-05 | 2 |
| 102 | TCGA-15-1444-01A-02D-1697-05 | 3 |
| 103 | TCGA-26-6174-01A-21D-1844-05 | 2 |
| 104 | TCGA-26-1442-01A-01D-1697-05 | 3 |
| 105 | TCGA-RR-A6KA-01A-21D-A33U-05 | 2 |
| 106 | TCGA-26-5132-01A-01D-1481-05 | 1 |
| 107 | TCGA-26-6173-01A-11D-1844-05 | 2 |
| 108 | TCGA-19-5947-01A-11D-1697-05 | 2 |
| 109 | TCGA-06-0171-01A-02D-A45W-05 | 2 |
| 110 | TCGA-06-1804-01A-01D-1697-05 | 1 |
| 111 | TCGA-76-6286-01A-11D-1844-05 | 1 |
| 112 | TCGA-RR-A6KB-01A-12D-A33U-05 | 2 |
| 113 | TCGA-06-6695-01A-11D-1844-05 | 1 |
| 114 | TCGA-32-1979-01A-01D-1697-05 | 2 |
| 115 | TCGA-26-5134-01A-01D-1481-05 | 3 |
| 116 | TCGA-19-A6J5-01A-21D-A33U-05 | 3 |
| 117 | TCGA-4W-AA9T-01A-11D-A392-05 | 2 |
| 118 | TCGA-OX-A56R-01A-11D-A33U-05 | 2 |
| 119 | TCGA-19-5954-01A-11D-1697-05 | 2 |
| 120 | TCGA-81-5911-01A-12D-1844-05 | 2 |
| 121 | TCGA-06-A7TL-01A-11D-A392-05 | 4 |
| 122 | TCGA-28-5209-01A-01D-1481-05 | 1 |
| 123 | TCGA-06-0221-01A-01D-A45W-05 | 2 |
| 124 | TCGA-06-6701-01A-11D-1844-05 | 3 |
| 125 | TCGA-12-5301-01A-01D-1481-05 | 3 |
| 126 | TCGA-76-4925-01A-01D-1481-05 | 1 |

**Table S15** | Pairwise log-rank comparisons of overall survival among the four molecular subtypes with Benjamini-Hochberg adjusted P values.

| Subtypes | | Adjusted_P |
| --- | --- | --- |
| 1 | 2 | 0.013 |
| 1 | 3 | 0.992 |
| 1 | 4 | 0.118 |
| 2 | 3 | 0.023 |
| 2 | 4 | 0.030 |
| 3 | 4 | 0.126 |

**Table S16** | Immune scores

| Sample | TIS | APM | CYT | GET | Stromal | Immune | ESTIMATE |
| --- | --- | --- | --- | --- | --- | --- | --- |
| TCGA-76-4927-01A | 3.02 | 11.71 | 0.91 | 0.54 | 204.79 | 259.06 | 463.85 |
| TCGA-28-5215-01A | 3.06 | 11.72 | 1.01 | 0.78 | 345.64 | 243.25 | 588.88 |
| TCGA-06-5416-01A | 2.81 | 11.73 | 0.65 | 0.41 | -1240.62 | -1096.39 | -2337.01 |
| TCGA-06-5408-01A | 2.95 | 12.12 | 0.82 | 0.57 | -570.04 | -352.08 | -922.12 |
| TCGA-12-5295-01A | 2.98 | 11.94 | 1.07 | 0.79 | -385.90 | 651.88 | 265.97 |
| TCGA-28-5213-01A | 3.10 | 11.95 | 1.18 | 0.83 | 739.83 | 1488.72 | 2228.55 |
| TCGA-06-5414-01A | 3.05 | 12.11 | 0.95 | 0.71 | -327.27 | 622.99 | 295.73 |
| TCGA-32-5222-01A | 2.99 | 11.92 | 0.87 | 0.46 | -223.38 | 583.11 | 359.73 |
| TCGA-28-5208-01A | 3.11 | 11.64 | 0.93 | 0.67 | -168.55 | 62.87 | -105.68 |
| TCGA-26-5133-01A | 2.86 | 11.72 | 0.74 | 0.35 | -867.78 | -924.41 | -1792.19 |
| TCGA-76-4932-01A | 2.82 | 11.92 | 0.76 | 0.42 | -876.01 | -122.99 | -999.00 |
| TCGA-06-5858-01A | 2.92 | 11.85 | 0.59 | 0.41 | -122.60 | 155.74 | 33.14 |
| TCGA-28-5218-01A | 3.33 | 11.41 | 1.32 | 1.21 | 1170.08 | 1007.50 | 2177.58 |
| TCGA-06-5413-01A | 3.21 | 11.77 | 1.22 | 0.83 | 310.43 | 433.55 | 743.98 |
| TCGA-15-1444-01A | 2.91 | 11.68 | 0.68 | 0.38 | -148.23 | 398.11 | 249.87 |
| TCGA-19-5960-01A | 2.76 | 11.29 | 0.65 | 0.44 | -1301.80 | -1279.04 | -2580.84 |
| TCGA-06-5415-01A | 3.02 | 11.97 | 0.90 | 0.53 | -774.08 | -670.47 | -1444.55 |
| TCGA-41-5651-01A | 2.89 | 11.17 | 0.81 | 0.35 | -938.68 | -1144.40 | -2083.07 |
| TCGA-28-5204-01A | 2.98 | 12.17 | 0.90 | 0.68 | -612.89 | -106.77 | -719.66 |
| TCGA-28-5207-01A | 3.09 | 11.66 | 0.87 | 0.63 | -47.68 | -245.42 | -293.10 |
| TCGA-19-4065-01A | 3.08 | 11.99 | 1.04 | 0.79 | 211.78 | 999.52 | 1211.30 |
| TCGA-06-1804-01A | 2.79 | 11.71 | 0.62 | 0.37 | -583.21 | -500.62 | -1083.83 |
| TCGA-06-0125-01A | 2.89 | 11.32 | 0.61 | 0.37 | -750.60 | -693.66 | -1444.25 |
| TCGA-32-1980-01A | 3.18 | 11.76 | 1.10 | 0.85 | 334.20 | 702.70 | 1036.90 |
| TCGA-06-0210-01A | 3.06 | 11.86 | 0.86 | 0.71 | -210.24 | 745.62 | 535.38 |
| TCGA-06-5411-01A | 3.03 | 11.36 | 0.74 | 0.53 | -346.62 | -136.84 | -483.46 |
| TCGA-76-4931-01A | 2.84 | 11.70 | 0.65 | 0.38 | -1094.32 | -765.45 | -1859.77 |
| TCGA-06-5410-01A | 3.32 | 12.08 | 1.12 | 1.03 | 948.32 | 2031.19 | 2979.50 |
| TCGA-06-5859-01A | 2.96 | 12.10 | 0.85 | 0.70 | -292.87 | 397.49 | 104.61 |
| TCGA-26-5132-01A | 2.99 | 12.05 | 0.87 | 0.68 | -465.24 | -164.84 | -630.08 |
| TCGA-28-5209-01A | 2.88 | 12.09 | 0.78 | 0.37 | -666.44 | -561.73 | -1228.17 |
| TCGA-26-1442-01A | 2.83 | 11.67 | 0.46 | 0.31 | -808.44 | -483.95 | -1292.39 |
| TCGA-28-5216-01A | 2.93 | 11.66 | 0.69 | 0.46 | -237.42 | -73.70 | -311.12 |
| TCGA-12-5299-01A | 2.94 | 11.91 | 0.71 | 0.47 | -412.62 | 142.01 | -270.60 |
| TCGA-06-6698-01A | 2.33 | 11.22 | 0.32 | 0.25 | -306.10 | 176.75 | -129.35 |
| TCGA-76-4929-01A | 2.81 | 11.79 | 0.59 | 0.37 | -481.84 | -354.12 | -835.97 |
| TCGA-06-5412-01A | 3.19 | 12.07 | 1.17 | 0.78 | 694.05 | 1346.57 | 2040.62 |
| TCGA-06-5417-01A | 2.65 | 11.58 | 0.54 | 0.26 | -885.39 | -756.88 | -1642.27 |
| TCGA-06-0190-01A | 3.22 | 12.03 | 1.17 | 0.97 | 649.35 | 814.58 | 1463.93 |
| TCGA-06-0211-01A | 3.13 | 11.93 | 1.05 | 0.84 | -43.21 | 543.00 | 499.79 |
| TCGA-76-4925-01A | 2.77 | 11.63 | 0.64 | 0.21 | -994.03 | -883.78 | -1877.81 |
| TCGA-26-5135-01A | 2.95 | 11.82 | 0.95 | 0.55 | -97.44 | 170.72 | 73.28 |
| TCGA-06-5418-01A | 2.89 | 11.92 | 0.74 | 0.42 | -395.99 | 396.80 | 0.80 |
| TCGA-28-2510-01A | 3.00 | 11.46 | 0.73 | 0.41 | -286.94 | -598.24 | -885.18 |
| TCGA-28-5220-01A | 2.85 | 11.74 | 0.82 | 0.48 | -576.36 | 98.28 | -478.08 |
| TCGA-26-5134-01A | 2.71 | 11.83 | 0.64 | 0.34 | -722.75 | -455.82 | -1178.57 |
| TCGA-26-5139-01A | 2.98 | 11.89 | 0.82 | 0.54 | -337.19 | 26.20 | -310.98 |
| TCGA-06-5856-01A | 3.04 | 11.76 | 0.91 | 0.52 | -209.80 | -27.27 | -237.07 |

**Table S17** | Genes used for CMap analysis

| Up-regulated | CCL18, IL2RA, LYVE1, F13A1, FPR2, CCL20, MARCO, CSF3, LRG1, VDR, CLDN23, CXCL8, RNF149, MRC1, COL13A1, NFKBIZ, CYP1B1, IGFBP6, MEDAG, RARRES1, FCGR2C, HSD11B1, MAP3K8, LILRB2, TNFAIP8, GPR171, CR1, IL7R, TFPI2, IL24, RAB11FIP1, CCDC71L, AL118508.1, TEC, KCNJ15, CD300E, LINC02301, ALDH1A3, PTGES, ZC3H12A, AC093627.1, VENTX, PI3, ETV3L, STEAP4, BIRC3, C5AR2, IER3, CCL7, CCL8, PTGER2, FAM83G, GPR141, MIR222HG, OR2I1P, AC116353.6, FAM20A, PTGS2, TNFAIP2, IL1R1, ACP3, EMB, PLB1, BANK1, SAA2, IL10, FCN1, CDCP1, RAB27A, ANPEP, RNASE3, COL6A3, CD200R1, AC020912.1, HK3, TMEM106A, JAML, B4GALT1, CSF2RB, AQP9, IGHV1-2, WDR45P1, CD209, JCHAIN, LILRA5, FAM177B, RNASE2, IGHA1, LINC00968, MAPK13, S100P, SPATA20P1, EREG, AL021978.1, TIMP1, CLEC2B, LTBP2, NAIP, AC243772.3, TSLP, SLC27A6, GCNT1, HGF, IL18R1, DMKN, DSE, LINC02742, CD300H, BAIAP2L1, MCTP2, TRPM2, IGKV3-11, AL590068.3, CD300LB, S100A9, AL355607.2, ARPC1B, LILRB3, SAA1, MAN1A1, FAM43A, ELF3, TREM1, AC083837.1, CCR2, LRRC37A11P, C1RL, TIMD4, CHI3L2, LYPD3, MIR3945HG, AC007877.1, CCL5, LINC01605, PTGER4, MYO1G, RBM47, GAPLINC, GPR160, PLAUR, SLC9A7P1, TXK, CTSW, THBD, MAFB, IL6, IGLC3, CRISPLD2, MCTP1, FMN1 |
| --- | --- |
| Down-regulated | LINC00689, ZNF560, FLJ16779, AL137017.1, AC020930.1, SUSD5, KLRC4-KLRK1, FERMT1, KLRC4, CYP27B1, AC098587.1, EPHB1, FREM3, AC104051.2, AC006042.2, LINC01098, KLRK1, AL118505.1, KLRC2, DLX5, LINC02283, PCDH15, UST, AL031668.2, RAC3, ADAMTS20, LINC01254, NEU4, GDNF-AS1, AC010425.1, AL512308.1, GOLGA6L2, NKAIN4, LINC01447, RPRM, AC083864.5, AL157700.1, AL136114.1, FRG1-DT, KLRC3, MARCHF9, CDK4, CRYAB, DCAF4L2, KRT5, LINC02223, ZNF727, MYCN, AP003100.2, SMCP, NPR3, AC006994.1, LIMS2, ZNF676, OR4K2, CRYBB2, AL033530.1, AL355306.2, PPDPFL, NR0B1, VN1R85P, AC107223.1, LINC01117, GPR17, WDR11-AS1, FRG2DP, KLRK1-AS1, ATP6V0A4, ATP13A5, HOXD3, DLL1, CSDC2, AC139530.1, LINC00906, NKD1, AL358473.1, LINC01694, LINC00652, LINC00588, ACRV1, AL606500.1, AC090398.2, LRRN4, AP005271.1, LINC02475, AC116362.1, AC069277.1, AC138761.1, CCND1, AL596257.1, SBK1, ZNF492, AC008991.1, AC131097.2, TLX2, ADAM29, MSX2, USP6, SOX8, AC006960.3, AP000842.3, ZNF679, NKX3-2, SRD5A2, RHBDL3, SIX6, VEPH1, AC007786.2, AC114316.1, AL645608.6, DSCAML1, FBXW12, CDH15, RCOR2, DEFB124, LINC02293, AC083805.2, CEROX1, AC073389.3, AL592211.2, ZNF849P, LHX1, PCDHA10, AL034550.3, AP000962.1, AL033539.2, LINC01088, PRSS35, NLRP11, RNA5SP18, AL512625.2, ALG1L8P, KIF21B, LINC02388, LINC02498, SYT6, FXYD2, AL136366.1, SNX22, ZNF488, AC020634.2, DQX1, NPSR1, PRKG2, PLAAT1, TRHR, LINC01385, AC020928.2, AL139231.1, GBP7 |
